# Functional and genomic profiling of lactic acid bacteria reveals specific traits as potential probiotics

**DOI:** 10.3389/fmicb.2026.1850174

**Published:** 2026-06-22

**Authors:** Inseo Kim, Sooyeon Kwon, Doyoon Shin, Soyun Choi, Eunkyung Choi, Hyun Park

**Affiliations:** Department of Biotechnology, College of Life Sciences and Biotechnology, Korea University, Seoul, Republic of Korea

**Keywords:** acid tolerance, antimicrobial activity, comparative genomics, intestinal adhesion, lactic acid bacteria, probiotics, whole-genome sequencing

## Abstract

The probiotic potential of lactic acid bacteria (LAB) and the subsequent long history of safe use in fermented foods and the human gastrointestinal tract are well-established. However, probiotic properties often vary considerably among species and strains, and the genomic determinants underlying these functional differences remain incompletely understood. Thus, this study obtained 27 LAB isolates from human oral cavity and cheese samples, and characterized these isolates to evaluate the associated probiotic potential using an integrative phenotypic and genomic approach. Molecular identification based on 16S rRNA gene sequencing assigned these isolates to five LAB species: *Lactobacillus rhamnosus*, *Lactobacillus curvatus*, *Lactobacillus reuteri*, *Lactococcus lactis*, and *Lactobacillus paracasei*, with *L. rhamnosus* being the most prevalent. *In vitro* assays revealed substantial variation among isolates in key probiotic traits, including acid and bile tolerance, adhesion to HT-29 intestinal epithelial cells, antimicrobial activity against pathogenic microorganisms, and antibiotic susceptibility. Notably, *L. rhamnosus* and *L. reuteri* exhibited the highest tolerance to acidic conditions and strong antimicrobial activities, while *L. reuteri* showed the greatest adhesion ability. Based on these phenotypic characteristics, seven representative strains were selected for whole-genome sequencing and comparative genomic analysis. Genomic characterization revealed genome sizes ranging from approximately 2.0–3.0 Mb and conserved functional gene categories related to metabolic processes and catalytic activity. Comparative analysis of adhesion-related genes identified expanded gene families (*galE*, *lgt*, *ftsW*, *ypeA*, *lytG*) linked to cell wall modification, lipoprotein maturation, and autolysin activity. Secondary metabolite predictions suggested a potential association between antimicrobial activity and species-level differences in RiPP-like and polyketide synthase clusters, including genes such as *mvaS, lagD*, LSEI_2163, and LSEI_2386. Overall, this study provides a comprehensive functional and genomic assessment of diverse LAB isolates and highlights specific genetic features associated with probiotic potential. These findings support the rational selection and development of LAB strains for next-generation probiotic applications.

## 1 Introduction

Lactic acid bacteria (LAB) are a diverse group of Gram-positive microorganisms widely distributed in fermented foods, the human gastrointestinal tract, and various environmental niches (Salvetti et al., 2012). Owing to the long history of safe use and well-documented health-promoting properties of LAB, these bacteria have been extensively investigated as probiotics, with reported benefits including modulation of the gut microbiota, enhancement of epithelial barrier integrity, inhibition of pathogenic microorganisms, and regulation of host immunity (Naidu et al., 1999; Sanders et al., 2010; Abouelela and Helmy, 2024). The functional attributes underlying these probiotic effects, such as acid and bile tolerance, adhesion to intestinal epithelial cells, production of antimicrobial compounds, and antibiotic resistance or susceptibility, are known to vary substantially at both the species and strain levels (Salomskiene et al., 2019; Jomehzadeh et al., 2020; Megur et al., 2023). Consequently, identifying LAB strains with robust probiotic potential and elucidating the genomic basis underlying these phenotypes is critical for the development of next-generation probiotics.

Advances in long-read sequencing technologies and comparative genomics have enabled high-resolution analyses of LAB genomes, yielding more accurate genome assemblies and annotations and facilitating detailed investigations into the genetic determinants of probiotic-related functions. In parallel, recent studies have increasingly emphasized integrating genomic data with phenotypic characterization to improve understanding of how genetic variation translates into functional differences (Li et al., 2020, 2023; Jiang et al., 2023). Indeed, by combining *in vitro* assays, such as tolerance to gastrointestinal stress, adhesion to intestinal epithelial cells, antimicrobial activity, and antibiotic susceptibility testing, with whole-genome and comparative genomic analyses, researchers have linked specific phenotypic traits to the associated underlying genetic determinants. Collectively, these integrative approaches have provided important mechanistic insights into how variations in metabolic pathways, regulatory networks, and cell surface-associated genes contribute to strain-dependent probiotic performance. However, most existing studies have focused on a limited number of strains or have been confined to a single LAB species, thereby limiting broader comparative insights into how probiotic traits and the associated genomic determinants vary across species. Therefore, systematic comparative investigations that evaluate multiple probiotic-related phenotypes and interpret the findings through comprehensive genomic analyses across diverse LAB species remain relatively limited. Consequently, further integrative studies encompassing diverse LAB isolates are required to deepen our understanding of the genomic foundations underlying probiotic potential and to support the rational selection of functionally robust LAB strains.

This study obtained 27 LAB isolates from human oral cavity and cheese samples, which were then examined for key probiotic traits, including acid and bile tolerance, adhesion to intestinal epithelial cells, antimicrobial activity, and antibiotic susceptibility. Based on the associated phenotypic profiles, seven representative strains were selected for whole-genome sequencing, followed by comparative genomic analyses focusing on amino acid metabolism, adhesion-associated genes, and secondary metabolite biosynthetic gene clusters. Thus, by integrating broad *in vitro* phenotypic characterization with comparative genomics across multiple LAB species, this study aimed to provide a more comprehensive understanding of the strain- and species-specific genetic features underlying probiotic potential in LAB.

## 2 Materials and methods

### 2.1 Identification of LAB by 16S rRNA gene sequencing

A total of 27 samples were collected from various environments, including the oral cavity and local cheese. All samples were cultured in de Man–Rogosa–Sharpe (MRS) broth (MB-M1025) and incubated at 37 °C for 24 h. Strain identification was performed by 16S rRNA gene sequencing (Beasley and Saris, 2004). This study used the polymerase chain reaction (PCR) primer pair 27F and 1492R: forward (27F), 5′–AGAGTTTGATCCTGGCTCAG–3′, and reverse (1492R), 5′–GGTTACCTTGTTACGACTT–3′ (Frank et al., 2008). PCR was performed in a final volume of 50 μL, containing 2 μL of template DNA, 1 μL of each primer, 25 μL of master mix, and 21 μL of terile distilled water. The PCR was conducted with the following program: an initial denaturation step of 95 °C for 5 min, followed by 35 cycles of 95 °C for 30 s, 55 °C for 30 s, and 72 °C for 2 min, with a final extension at 72 °C for 5 min. Then, 6 μL of each PCR product was separated by electrophoresis on a 1.0% (w/v) agarose gel. The resulting 1,465 bp amplicons were purified using a Gel and PCR Clean-up kit (Cosmo, Seoul, Korea) and sequenced by Macrogen. The sequencing results were compared with database entries using the Basic Local Alignment Search Tool (BLAST).^1^

### 2.2 Assessment of *in vitro* characteristics

#### 2.2.1 Acid and bile tolerance

Acid tolerance of the isolates was tested as previously described, with minor modifications (Jomehzadeh et al., 2020). Briefly, acidified MRS broth was prepared by adjusting the pH to 4.0 using 5 N hydrochloric acid (HCl). All isolates were first cultured in MRS broth and incubated at 37 °C for 24 h. An overnight culture was then inoculated into standard MRS broth and MRS broth at pH 4.0 and incubated anaerobically at 37 °C for 2 h. After incubation, the number of surviving bacteria was measured by spreading onto MRS agar plates, which were then incubated at 37 °C for 24 h. The survival rate (%) was calculated by comparing the number of viable cells using the following formula:


$$\mathrm{Survival}\mathrm{rate}\left(\%\right)=N/N_{0}\times 100\%$$

where N_0_ is the total number of viable bacteria in the untreated group, and N is the total number of viable bacteria after treatment.

The bile tolerance assay was performed as previously described, with minor modifications (Jomehzadeh et al., 2020). Bile tolerance was examined in MRS broth supplemented with 0.3% (w/v) oxgall bile salts (Sigma-Aldrich Co., St. Louis, MO, United States). All samples were cultured in MRS broth and incubated at 37 °C for 24 h. An overnight culture of LAB strains (10^8^ CFU/mL) was inoculated into MRS broth with or without 0.3% (w/v) bile salts and incubated anaerobically at 37 °C for 8 h. After incubation, the survival rate (%) was calculated by comparing the OD600 value of surviving cells after incubation (OD) with the OD600 value of the initial culture (OD_0_) according to the following formula:


$$\mathrm{Survival}\mathrm{rate}\left(\%\right)=\mathrm{OD}/{\mathrm{OD}}_{0}\times 100\%$$

#### 2.2.2 Adhesion assay

Adhesion to human epithelial cells was assessed as previously described (Verdenelli et al., 2009). The ability of lactic acid bacterial isolates to adhere to human epithelial cells was examined using the HT-29 cell line. HT-29 cells were cultured in Dulbecco’s modified Eagle’s medium (DMEM) supplemented with 10% (v/v) fetal bovine serum (FBS), penicillin–streptomycin (100X), 100 mM sodium pyruvate, 1 M HEPES, and 2-mercaptoethanol (1,000X), and maintained in a controlled atmosphere of 5% CO_2_ at 37°C.

For the bacterial adhesion assay, HT-29 cell monolayers were seeded in 6-well tissue culture plates and incubated at 37 °C in 5% CO_2_ atmosphere for 48 h. After incubation, the monolayers were washed twice with Dulbecco’s phosphate-buffered saline (DPBS), and 1 mL of bacterial suspension (10^8^ CFU/mL) was added to each well. The inoculated plates were then incubated at 37 °C in 5% CO_2_ for 2 h. Subsequently, the plates were washed three times with DPBS to remove non-adherent bacteria. The adherent bacteria were detached from the wells using trypsin–EDTA (0.05%) and washed in DPBS. Then, adherent bacteria were resuspended in MRS broth and cultured on MRS agar. After anaerobic incubation at 37 °C for 48 h, the percentage of adherent bacteria was determined by comparing the number of adhered cells (A) with the total number of cells in the initial bacterial suspension (A_0_), according to the following formula:


$$\mathrm{Adherent}\mathrm{rate}\left(\%\right)=A/A_{0}\times 100\%$$

#### 2.2.3 Antimicrobial activity

The antimicrobial activity of lactic acid bacterial strains against selected indicator microorganisms was further assessed using a broth-based assay measuring the OD600 of bacterial suspensions in the presence and absence of cell-free culture supernatants (CFCS) (Balouiri et al., 2016). The CFCS were prepared by growing cultures of lactic acid bacterial strains in MRS broth at 37 °C for 24 h, then centrifuging the cultures at 3,000 rpm for 10 min. Bacterial strains, including *Staphylococcus aureus* and *Escherichia coli*, were inoculated in Luria–Bertani (LB) broth, and *Candida albicans* was inoculated in glucose–peptone–yeast extract agar (GPYA) broth. After overnight incubation at 37 °C, 900 μL of the CFCS was inoculated into each bacterial suspension, and the bacterial suspensions with and without CFCS were incubated at 37 °C for 5 h. The survival rate was defined as the ratio of the OD600 of surviving cells after CFCS treatment (OD_*c*_) to the OD600 value of the untreated control (OD_*o*_), according to the following formula:


$$\mathrm{Antimicrobial}\mathrm{activity}\left(\%\right)={\mathrm{OD}}_{c}/{\mathrm{OD}}_{o}\times 100\%$$

#### 2.2.4 Antibiotic susceptibility assay

Antibiotic susceptibility was assessed using the disk diffusion method (Rajoka et al., 2018). LAB strains were inoculated in MRS broth and incubated at 37 °C for 24 h. The isolated LAB strains were tested for susceptibility to six antimicrobial agents: penicillin (10 units), vancomycin (30 μg), cephalothin (30 μg), gentamicin (10 μg), chloramphenicol (30 μg), and tetracycline (30 μg). After incubation at 37 °C for 24 h, the inhibition zone diameters were measured for each sample, and antibiotic susceptibility was classified as susceptible, moderately susceptible, or resistant. Bacterial sensitivity was determined according to the Clinical and Laboratory Standards Institute (CLSI) guidelines (Supplementary Table 1; Reller et al., 2009; Weinstein and Lewis, 2020).

### 2.3 Whole genome sequencing

High-quality genomic DNA was sheared to fragments > 15 kb using a Covaris g-TUBE (Covaris, USA) and purified with 0.45 × AMPure XP magnetic beads (Beckman Coulter, United States). Fragment size distribution was assessed using a Bioanalyzer (Agilent Technologies, United States). The sheared DNA was then incubated with NAD^+^, DNA prep buffer, DNA prep enzyme, and DNA prep additive at 37 °C for 15 min to remove single-stranded overhangs. DNA damage repair was performed by adding DNA Damage Repair Mix and incubating at 37 °C for 30 min, followed by end-repair using End Repair Mix at 20 °C for 10 min and 65 °C for 30 min. Subsequently, an overhang adapter was ligated to the repaired DNA fragments by adding adapter, ligation mix, additive, and enhancer, followed by incubation at 20 °C for 60 min and 65 °C for 10 min. Residual unligated products were removed using the Enzyme Cleanup kit at 37 °C for 1 h. The final library was purified with 0.45 × AMPure XP beads, and its concentration and fragment size were verified using a Bioanalyzer. Libraries were size-selected using a BluePippin system (Sage Science, United States) with a 0.75 % gel cassette, collecting fractions larger than 9–13 and 15 kb (BP start: 9,000 bp; BP end: 13,000 bp). The size-selected library was further purified with 0.5 × AMPure XP beads, quantified, and bound to magnetic beads (MagBead) for sequencing on the PacBio Sequel platform using a Sequel SMRT Cell 8M.

*De novo* genome assembly was performed using Flye v2.8.3 with PacBio subreads (Kolmogorov et al., 2019). If assembly quality was suboptimal, HGAP4 or NextDenovo v2.4.0 was used instead, and the best assembly result was selected (Chin et al., 2013; Hu et al., 2024). For bacterial genomes, Circlator v1.5.5 was used to determine circularization and to rotate the genome so that the *dnaA* gene was positioned at the start coordinate (Hunt et al., 2015). Gene prediction was conducted using Prokka, and functional annotation of predicted genes was carried out with Blast2GO based on Gene Ontology (GO) terms and sequence similarity searches (Conesa et al., 2005; Seemann, 2014).

### 2.4 Comparative genomic analysis

For comparative genomic and phylogenetic analyses, genomic data for other LAB strains were retrieved from the National Center for Biotechnology Information (NCBI) database (Supplementary Table 2). Orthologous gene families were identified using OrthoFinder (v2.5.4) with protein-coding genes from the experimental LAB strains, reference strains, and outgroup species (Emms and Kelly, 2019). Gene counts derived from the OrthoFinder output were merged and used to calculate the mean, standard deviation, and Z-scores; gene families with Z-scores ≥ 2 or ≤−2 were classified as expanded and contracted, respectively. These data were then used in CAFE (Computational Analysis of gene Family Evolution) analysis to assess gene family evolution (De Bie et al., 2006). A phylogenetic tree was constructed in MEGA-X using the maximum-likelihood (ML) method, based on 98,035 amino acid positions from concatenated single-copy orthologous proteins (Kumar et al., 2018). Statistical support for each node was evaluated using 1,000 bootstrap replications with the built-in bootstrap method.

Lactic acid bacteria cell adhesion-related proteins previously reported in the literature were collected, and the associated reference sequences were retrieved from NCBI. The presence of these adhesion proteins in the seven LAB isolates was assessed using the BV-BRC (v3.54.6) BLASTp tool, and heatmaps were generated from the bit-scores of the top hits (Olson et al., 2023). In addition, the genomes of the LAB isolates were screened for genes encoding putative antimicrobial compounds using BAGEL4 (van Heel et al., 2018) and antiSMASH v8.0 (Blin et al., 2025).

### 2.5 Statistical analysis

All experimental data were obtained from three independent replicates and are presented as the mean ± standard deviation (SD). Statistical significance (*p* < 0.05) was determined by one-way analysis of variance (ANOVA) with Tukey’s *post-hoc* test. All statistical analyses were performed using Python (SciPy v1.17.1 and statsmodels v0.14.6) (Wang et al., 2022).

## 3 Results

### 3.1 Molecular identification of LAB by 16S rRNA sequencing

A total of 27 isolates were positive for the target 16S rRNA fragment by PCR. The isolates were identified by sequencing the 16S rRNA gene. In total, 27 isolates were identified as *L. rhamnosus* (*n* = 11), *L. curvatus* (*n* = 4), *L. reuteri* (*n* = 4), *Lc. lactis* (*n* = 4), and *L. paracasei* (*n* = 4). These results indicate that *L. rhamnosus* is the dominant strain in the oral cavity, and that cheese harbors four lactic acid bacterial species: *L. curvatus*, *L. reuteri*, *Lc. lactis*, and *L. paracasei* (Supplementary Table 3).

### 3.2 Assessment of *in vitro* characteristics

#### 3.2.1 Probiotic survival in the gastrointestinal tract conditions

The acid tolerance profiles of various LAB strains are summarized in Supplementary Table 4. The number of viable cells was converted to log values (log CFU/mL). Both *L. rhamnosus* and *L. reuteri* exhibited high survival rates of 98.68 ± 7.63% and 98.22 ± 6.34%, respectively, after exposure to pH 4.0. In contrast, *L. curvatus, Lc. lactis*, and *L. paracasei* did not survive at pH 4.0, although the *Lc. lactis* SGL30066 (83.06 ± 2.58%) and *L. paracasei* SGL30088 (107.33 ± 1.07%) strains remained viable.

All lactic acid bacterial isolates capable of withstanding acidic conditions were then exposed to 0.3% bile for 8 h. The results indicated that more than 50% of the initial cell population survived, suggesting that all tested strains were tolerant to bile conditions.

The adhesion assay results are summarized in Supplementary Table 4. These results indicate that the isolates can adhere to HT-29 epithelial cells. Among the tested strains, the highest level of adherence was observed for *L. reuteri* (80.58 ± 1.82%); meanwhile, *L. rhamnosus*, *L. curvatus, Lc. lactis*, and *L. paracasei* exhibited comparable adhesive capacities, with adherence rates of 49.24 ± 0.97%, 52.05 ± 7.70%, 54.63 ± 6.52%, and 64.00 ± 7.00%, respectively. In contrast, *Lc. lactis* SGL30066 did not adhere to HT-29 cells.

#### 3.2.2 Antimicrobial activity

The antibacterial activity of all tested strains is summarized in Supplementary Table 5. Notably, *L. rhamnosus* showed the strongest antimicrobial activity against *E. coli*, *S. aureus*, and *Candida albicans*. *L. reuteri* and *L. curvatus* inhibited the growth of *S. aureus* more effectively than *E. coli* and *C. albicans*, whereas *L. paracasei* showed stronger antibacterial activity against *C. albicans* than against the other pathogens.

#### 3.2.3 Antibiotic susceptibility assay

The susceptibility patterns of LAB strains to six antibiotics are shown in Supplementary Table 6. For penicillin, only the *Lc. lactis* SGL30067 strain exhibited intermediate resistance, while all other strains were resistant. Additionally, *Lc. lactis* were sensitive to vancomycin, whereas all other strains were resistant. Among the tested strains, *L. reuteri, L. curvatus*, and *Lc. lactis* were susceptible to cephalothin, and *L. rhamnosus* and *L. curvatus* were susceptible to tetracycline. Most LAB were susceptible to chloramphenicol, except for the *Lc. lactis* SGL30067 and *L. paracasei* SGL30091 strains. All tested strains were resistant to gentamicin.

### 3.3 Genomic characteristics of LAB

**FIGURE 1:**
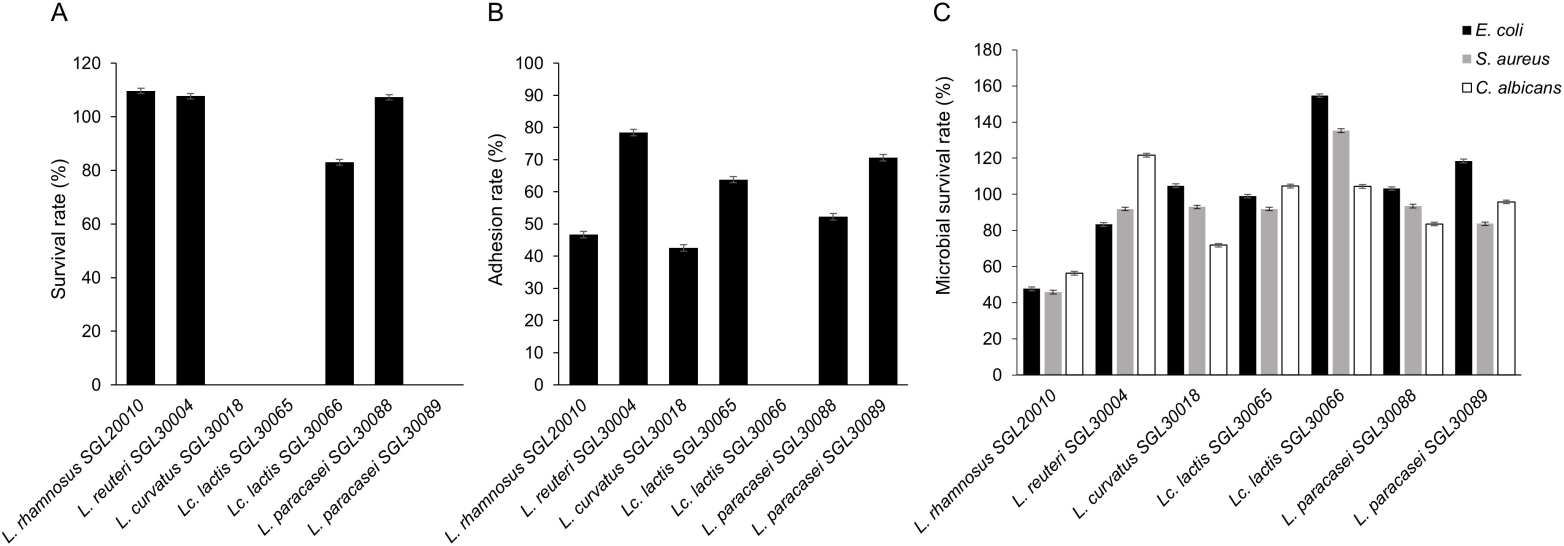
Phenotypic characterization of selected lactic acid bacterial strains. Phenotypic evaluation of seven lactic acid bacterial strains selected for genomic analysis. **(A)** Acid tolerance of the strains after exposure to acidic conditions (pH 4.0) at 37 °C for 2 h. Survival rates were calculated based on colony-forming unit (CFU) counts before and after acid exposure. **(B)** Adhesion ability of the strains to HT-29 human intestinal epithelial cells. Adhesion efficiency is expressed as the percentage of bacteria adhering to the cells relative to the initial inoculum. **(C)** Antimicrobial activity against indicator pathogenic bacteria, assessed with cell-free culture supernatants. Data are presented as the mean ± standard deviation from three independent experiments.

**FIGURE 2:**
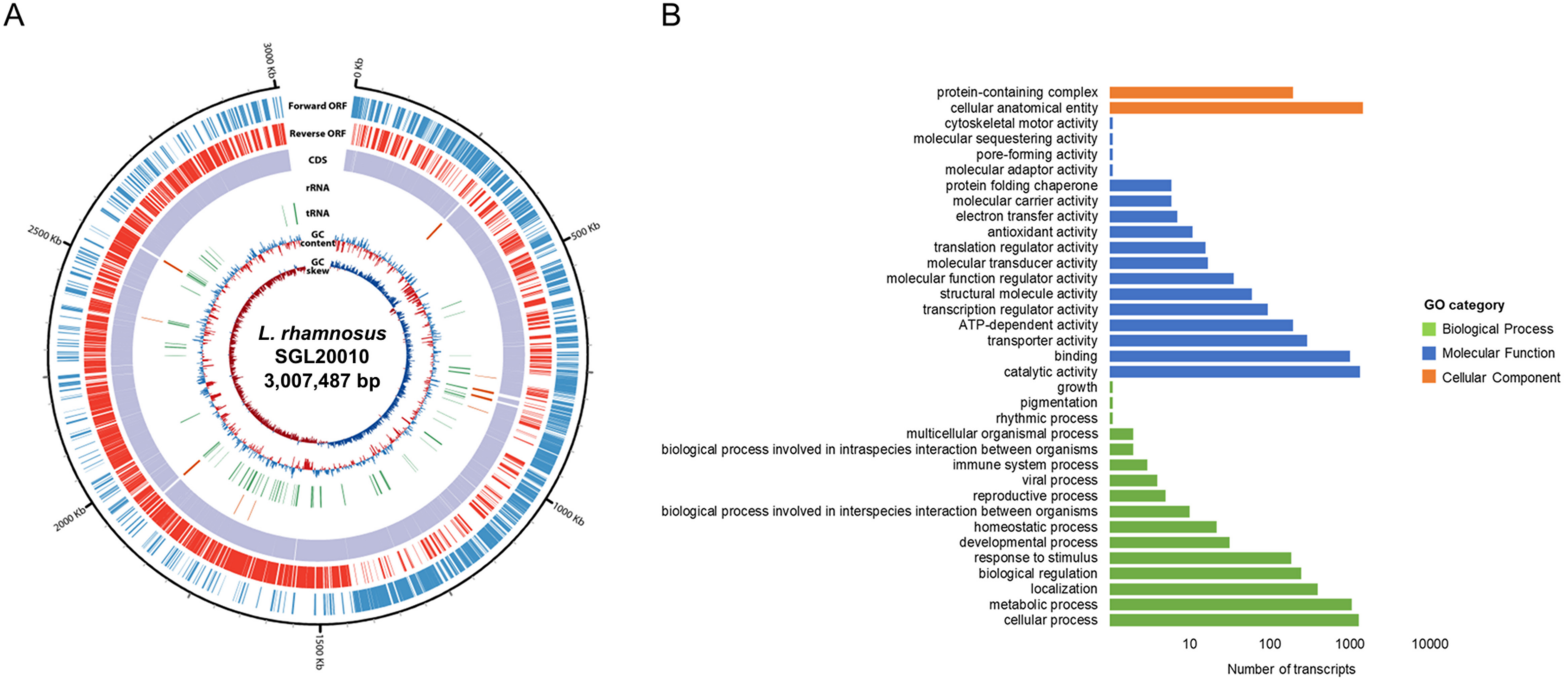
Genomic features and functional annotation of selected lactic acid bacterial strains. **(A)** Circular genome map (Circos plot) of the representative strain *L. rhamnosus* SGL20010 showing the distribution of coding sequences (CDS), GC content, and GC skew across the chromosome. Genes located on the forward and reverse strands are displayed in separate tracks. **(B)** Gene Ontology (GO)-based functional classification of predicted genes in *L. rhamnosus* SGL20010. Annotated genes were categorized into the three main GO domains: biological process (BP), molecular function (MF), and cellular component (CC). The most abundant functional categories included metabolic process and cellular process in BP, catalytic activity and binding in MF, and cellular anatomical entity in CC.

Strains were selected for whole-genome sequencing based on the *in vitro* characterization results. In principle, representative strains were selected for each species based on overall probiotic performance; however, two additional acid-tolerant strains (SGL30066 and SGL30088) were included for *Lc. lactis* and *L. paracasei* to examine strain-level differences. In total, seven strains were selected for genome sequencing (Figure 1). Details of the assembly and annotation statistics for each genome are presented in Table 1. The basic genomic features of the LAB strains were statistically evaluated. The average genome length of the seven strains was 2,593,732 bp, with *L. rhamnosus* SGL20010 possessing the largest genome (3.01 Mb) (Figure 2A). For clarity, the Circos map is shown for a representative strain, while the corresponding genomic maps for the remaining strains are provided in Supplementary Figure 1. The mean GC content was 41.59%, and both *L. rhamnosus* and *L. paracasei* exhibited relatively higher GC contents than the other strains. Consistently, *L. rhamnosus* SGL20010 had the greatest number of CDS (2,815), whereas *L. curvatus* SGL30018 exhibited the fewest (1,953).

**TABLE 1:** Genomic characteristics of lactic acid bacterial strains.

Strain	Species	Genome size (bp)	GC content (%)	N50 (bp)	No. of CDS	No. of rRNA	No. of tRNA
SGL20010	*L. rhamnosus*	3,007,487	46.69	3,007,487	2,815	15	59
SGL30004	*L. reuteri*	2,099,295	38.81	2,074,356	2,015	18	69
SGL30018	*L. curvatus*	1,912,649	42.04	1,912,649	1,953	18	65
SGL30065	*Lc. lactis*	2,600,442	35.16	2,513,880	2,527	19	64
SGL30066	*Lc. lactis*	2,689,878	35.16	2,513,887	2,527	19	64
SGL30088	*L. paracasei*	2,923,203	46.63	2,787,985	2,749	15	60
SGL30089	*L. paracasei*	2,923,169	46.63	2,787,973	2,749	15	60

**FIGURE 3:**
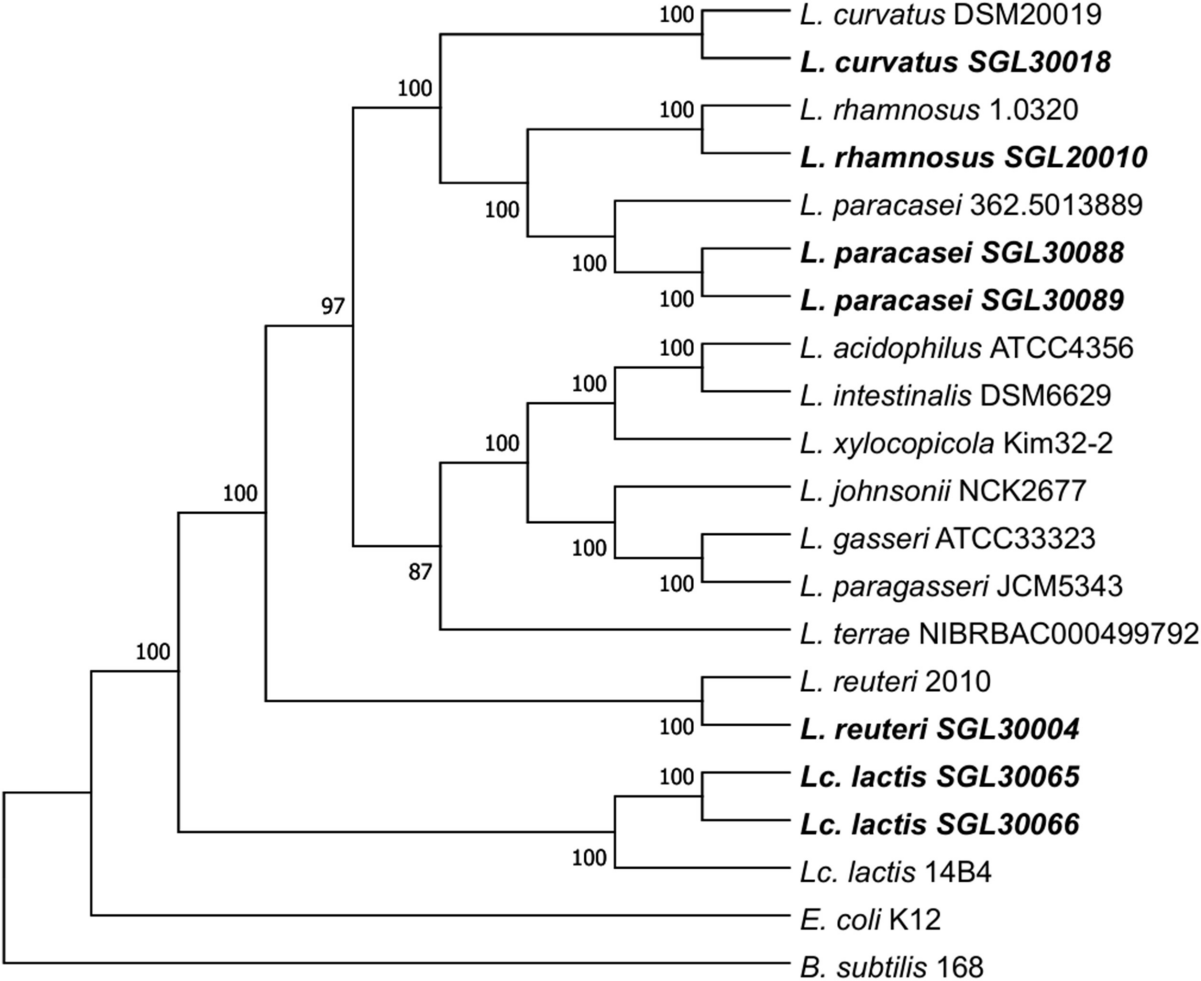
Maximum-likelihood phylogenetic tree inferred from concatenated alignments of single-copy orthologous proteins from the seven analyzed lactic acid bacterial strains and their related strains. The tree was constructed using the JTT matrix-based model, and node support values were estimated from 1,000 bootstrap replicates. The strains clustered into distinct clades consistent with their species-level taxonomic classification.

In the Gene Ontology (GO) analysis, all seven strains showed similar functional distributions: the highest numbers of transcripts were assigned to catalytic activity and binding in the molecular function (MF) category; cellular anatomical entity in the cellular component (CC) category; cellular process and metabolic process in the biological process (BP) category (Figure 2B). GO profiles for the remaining strains are presented in Supplementary Figure 2. Phylogenetic relationships were inferred using the ML approach based on the concatenated alignment of single-copy orthologous proteins. The resulting ML tree clearly resolved the strains into distinct clades corresponding to the associated species-level classifications (Figure 3). Bootstrap values from 1,000 replicates provided strong support for the major branching patterns.

### 3.4 Comparative genomic analysis

#### 3.4.1 Identification of acid tolerance genes

The *in vitro* characterization assays revealed that *L. rhamnosus* and *L. reuteri* exhibited the highest acid tolerance, whereas *L. curvatus* showed no tolerance under the same conditions. Among the *Lc. lactis* and *L. paracasei* isolates, only strains SGL30066 and SGL30088, respectively, represented acid resistance. Based on these phenotypic differences, the genomic basis underlying the variation in acid tolerance across species was investigated. Since exposure to acidic environments represents a major form of cellular stress, previous studies have consistently suggested that acid tolerance is closely linked to the presence and activity of stress-response genes (Li et al., 2020).

Indeed, stress-tolerance genes are known to play key roles in maintaining protein stability, protecting cellular structures, and regulating intracellular pH under harsh conditions. Molecular chaperones such as GroEL/GroES and DnaK refold or stabilize proteins that become denatured during stress, whereas Clp proteases remove damaged proteins to preserve cellular homeostasis (Ferreira et al., 2013; Hamon et al., 2014). Proton-pumping systems, including the F_0_F_1_–ATPase complex, directly contribute to acid resistance by exporting excess protons from the cytoplasm (Sánchez et al., 2006). Additional genes involved in oxidative stress detoxification and cell envelope reinforcement further enhance survival in acidic and other stressful environments (Wu et al., 2013; Paradeshi et al., 2018).

**FIGURE 4:**
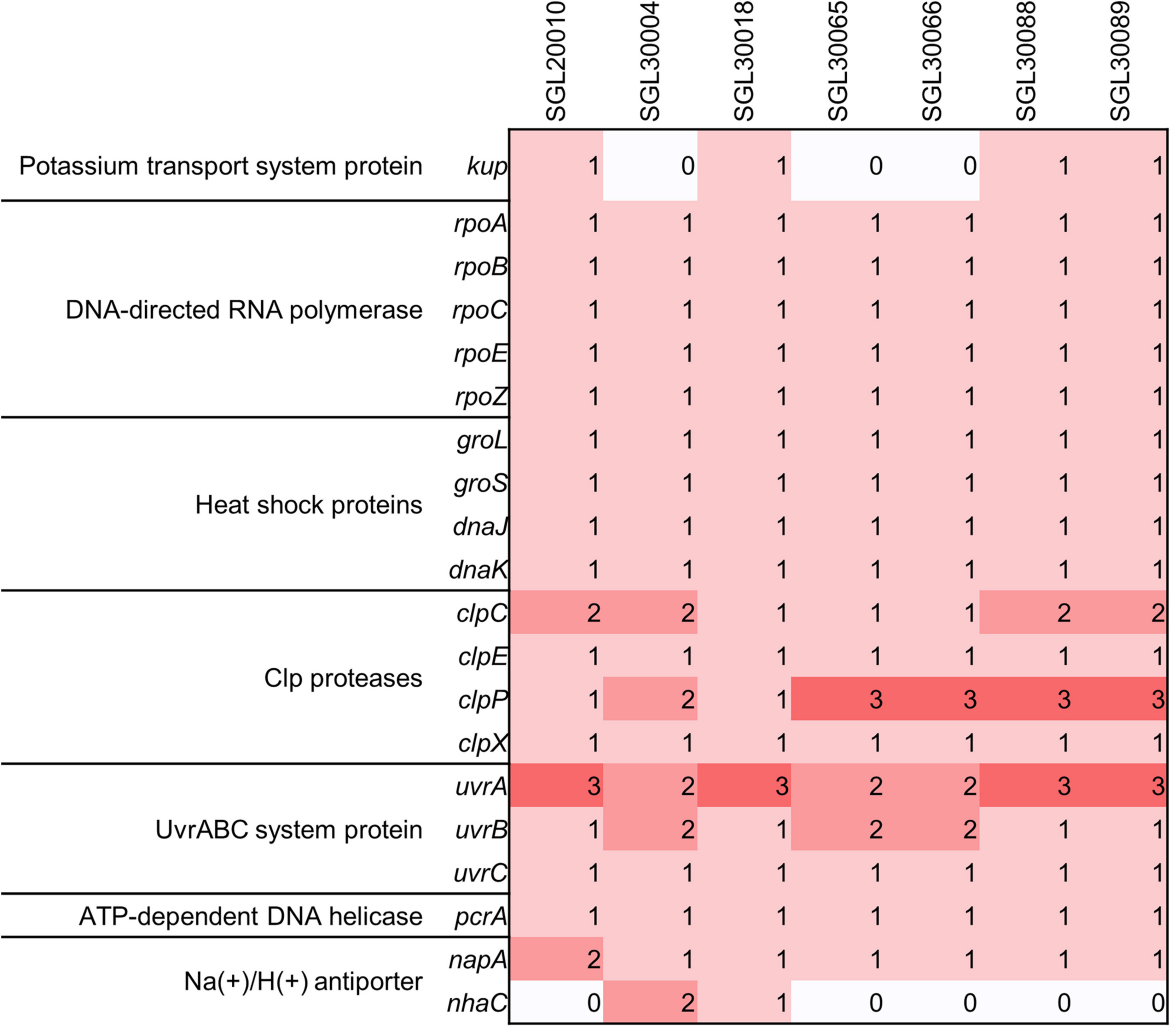
Heatmap of stress tolerance-associated gene counts in lactic acid bacterial strains. The heatmap shows the distribution and abundance of genes associated with general stress tolerance across seven sequenced lactic acid bacteria (LAB) genomes. Gene counts were derived from genome annotations and functional classifications. Color intensity represents the relative number of genes detected for each functional category in each strain.

**FIGURE 5:**
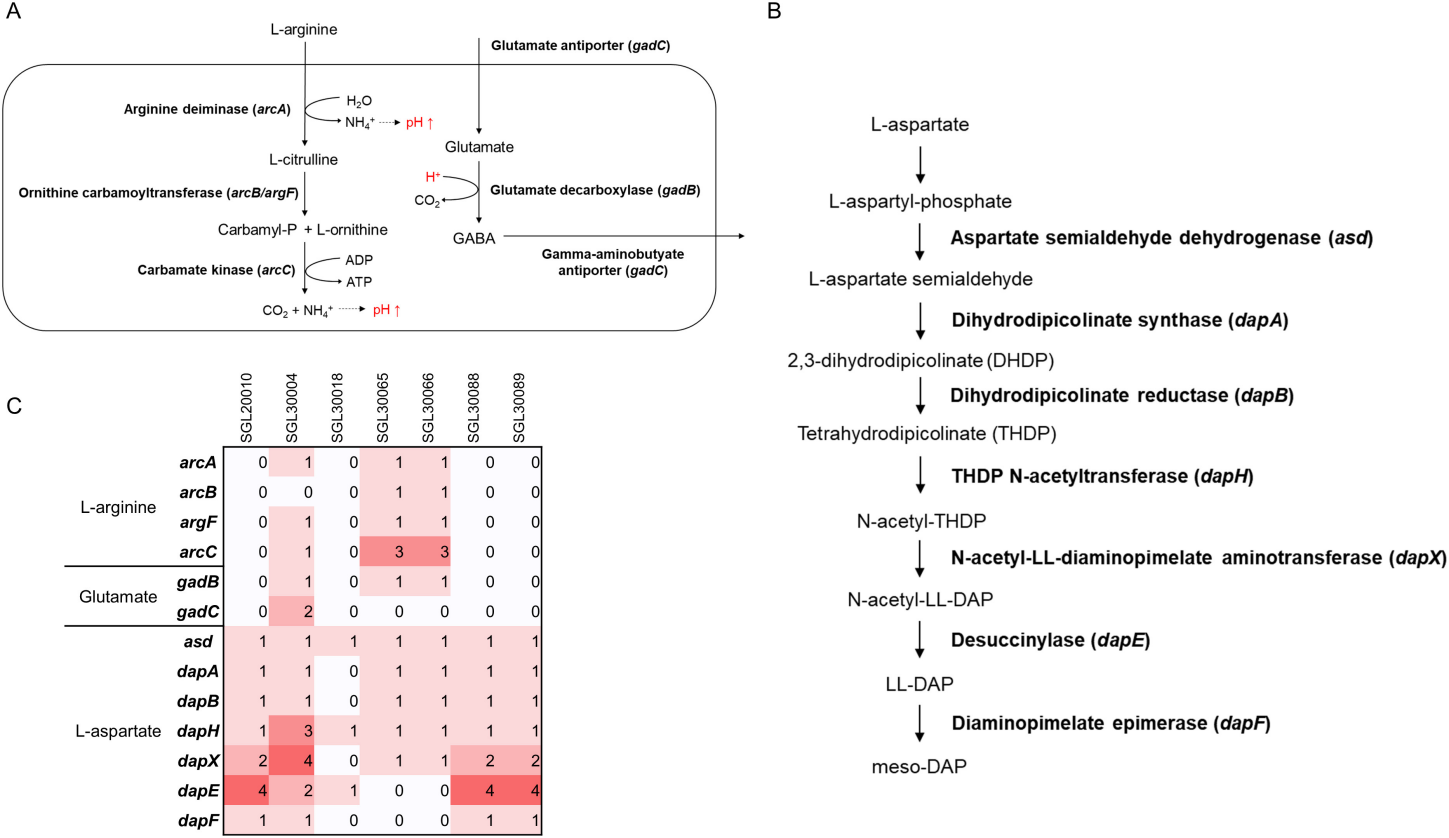
Amino acid metabolism pathways associated with acid tolerance in lactic acid bacteria (LAB). **(A)** Schematic representation of amino acid degradation pathways contributing to acid resistance in LAB, such as L-arginine and glutamate metabolism, which generate ammonia and help maintain intracellular pH. **(B)** Biosynthetic pathway converting L-aspartate to meso-diaminopimelate (meso-DAP), an important precursor for peptidoglycan synthesis that enhances cell wall stability under acidic conditions. **(C)** Heatmap of the presence or absence of genes involved in amino acid metabolism pathways related to acid tolerance in the seven analyzed genomes of LAB.

Gene count analysis of stress tolerance-related genes revealed no major differences between acid-tolerant and non-acid-tolerant strains, indicating that the presence of these general stress-response genes alone does not fully explain the observed phenotypic variation (Figure 4). This prompted the examination of genes involved in amino acid metabolism as alternative candidates for acid tolerance. Recent studies have shown that amino acid metabolism in LAB contributes to several physiological processes, including intracellular pH regulation, energy and redox generation, and resistance to environmental stresses (Zhang et al., 2012; Reeve and Reid, 2016). In particular, the catabolism of L-arginine and glutamate consumes protons and generates ammonia, both of which support acid resistance (Figure 5A; Zhang et al., 2012; Park et al., 2021). L-aspartate metabolism also enhances tolerance by producing meso-diaminopimelate (meso-DAP), a key precursor for peptidoglycan cross-linking that strengthens the cell wall as a physical barrier under acidic conditions (Figure 5B; Xu et al., 2019). Based on these insights, the seven LAB genomes were examined for the presence of amino acid metabolism-related genes. The non-acid-tolerant *L. curvatus* strain SGL30018 possessed markedly fewer of these genes than the other strains, particularly those involved in the conversion of L-aspartate to meso-DAP (Figure 5C). This pattern suggests that amino acid metabolic pathways, rather than general stress-tolerance genes, may play a more prominent role in determining acid tolerance in LAB. In addition, all lactic acid bacterial strains, except SGL30004, SGL30065, and SGL30066, lacked genes directly involved in the L-arginine and glutamate degradation pathways. Further analysis revealed that all strains possessed multiple aminopeptidase genes associated with amino acid catabolism, including *pepN*, *pepC*, *pepE*, *pepT*, and *pepV* (Liu et al., 2010). Genes involved in amino acid transport (*artP*, *artQ*, *artM*, *gltT*, and *yveA*) were also present across all strain genomes (Supplementary Table 7). These findings suggest that, even in the absence of specific amino acid catabolic pathways directly linked to acid tolerance, the strains retain the capacity to import and utilize amino acids through alternative aminopeptidase activities and transport systems, providing a supplementary mechanism that may contribute to cellular survival under acidic stress.

#### 3.4.2 Analysis of gene-encoded cell adhesion proteins

**FIGURE 6:**
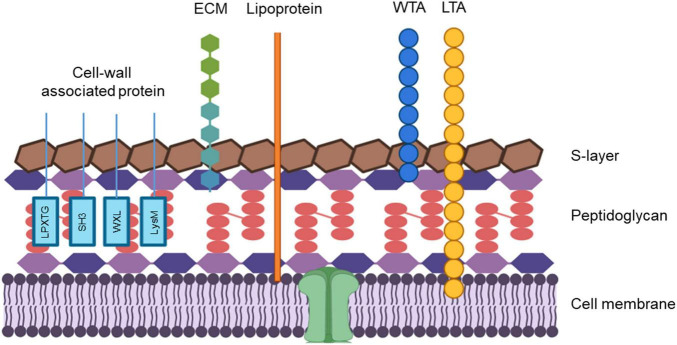
Cell surface structure of Gram-positive bacteria relevant to adhesion and host interactions. The cell membrane is enclosed by a multilayered peptidoglycan shell decorated with lipoteichoic acids (LTAs), wall teichoic acids (WTAs), surface proteins, and lipoproteins. Exopolysaccharides (EPS) form a compact layer closely associated with the peptidoglycan, which is further overlaid by an outer envelope of S-layer proteins. Membrane proteins can covalently attach to the long-chain fatty acids of the cytoplasmic membrane. Proteins are attached to the cell wall either covalently (LPXTG proteins) or non-covalently (via LysM, SH3, or WXL domains) or are lipid-anchored to the cell membrane (lipoproteins).

Lactic acid bacteria, such as other Gram-positive bacteria, possess a cytoplasmic membrane, a thick cell wall, and various surface structures. The cytoplasmic membrane comprises a phospholipid bilayer, from which teichoic acids—lipoteichoic acid (LTA) and wall teichoic acid (WTA)—extend to interact with the peptidoglycan layer. Above the membrane, a thick peptidoglycan layer is formed, to which proteins are anchored through different mechanisms: LPXTG proteins are covalently linked to peptidoglycan by sortase enzymes, whereas the SH3, WXL, and LysM proteins are attached non-covalently. Additionally, lipoproteins are covalently attached to membrane lipids (Figure 6; Liu et al., 2010).

Using CAFE analysis, expanded and contracted gene families were identified across lactic acid bacterial strains, and the expanded genes were subsequently selected for their association with adhesion ability. Notably, five adhesion-related genes were identified across four strains: in strain SGL30004, the gene lytG, which encodes an exo-glucosaminidase, was expanded; in strain SGL30018, galE, which encodes UDP-glucose 4-epimerase, and lgt, which encodes phosphatidylglycerol prolipoprotein diacylglyceryl transferase, were expanded; in strains SGL30065 and SGL30066, ftsW, which encodes a putative peptidoglycan glycosyltransferase, and ypeA, which encodes an acetyltransferase, were expanded (Table 2). The proteins encoded by these genes participate in adhesion through three major functional stages: (i) preparation of the cell wall and surface structures, (ii) display of surface proteins and lipoprotein adhesion factors, and (iii) promotion of cell surface attachment (Bos et al., 1999).

**TABLE 2:** Expanded genes associated with the adhesion ability of lactic acid bacterial strains.

Strain	Gene	Description
SGL30004	*lytG*	Exo-glucosaminidase LytG
SGL30018	*galE*	UDP-glucose 4-epimerase
	*lgt*	Phosphatidylglycerolprolipoprotein diacylglyceryl transferase
SGL30065 SGL30066	*ftsW*	Putative peptidoglycan glycosyltransferase FtsW
	*ypeA*	Acetyltransferase YpeA

In the initial stage, which involves preparation of the cell wall and surface structures, the expanded genes include *galE* in SGL30018 and *ftsW* and *ypeA* in SGL30065 and SGL30066. UDP-glucose 4-epimerase (GalE) functions as a key enzyme that interconverts UDP-glucose and UDP-galactose, thereby supplying precursors for exopolysaccharides (EPS) and teichoic acids located on the outer cell wall (Iskandar et al., 2019). EPS contributes to adhesion by modulating surface charge and hydrophobicity (Pramudito et al., 2024). The peptidoglycan glycosyltransferase FtsW performs transglycosylation by polymerizing lipid II and elongating the peptidoglycan chain; changes in peptidoglycan thickness or porosity can alter the exposure of surface proteins and EPS, thereby influencing adhesive interactions (Yuan et al., 2007). The acetyltransferase encoded by *ypeA* may modify the acetylation state of surface polysaccharides, including EPS, thereby affecting charge distribution and hydrogen-bonding properties that are closely associated with bacterial adhesion (Sengupta et al., 2013). Thus, these genes act upstream in the adhesion pathway by shaping the structural and biochemical properties of the cell envelope.

In the intermediate stage, where surface proteins and lipoprotein adhesion factors are displayed, the expanded gene *lgt* (phosphatidylglycerol prolipoprotein diacylglyceryl transferase) in SGL30018 plays a central role. This enzyme catalyzes the first step in lipoprotein maturation, and lipoproteins serve as key surface factors in Gram-positive bacteria, contributing to adhesion, nutrient acquisition, and cell wall stability (Sengupta et al., 2013). Loss of *lgt* has been shown to reduce adhesion and invasiveness in several Gram-positive organisms, highlighting the associated functional importance (Baumgärtner et al., 2007).

The final stage, which centers on the direct promotion of adhesion, is represented by the expanded *lytG* gene in SGL30004. Peptidoglycan is composed of alternating GlcNAc and MurNAc units, and exo-glucosaminidase functions as a cell wall hydrolase (autolysin) that cleaves GlcNAc residues (Müller et al., 2021). Such cell wall remodeling alters the exposure patterns of surface proteins and lipoproteins, modifies surface charge and hydrophobicity, and thereby affects adhesion capacity. Moreover, autolysin activity releases eDNA (extracellular DNA) and peptidoglycan fragments, which act as adhesive components within the biofilm matrix and promote biofilm formation (Panlilio and Rice, 2021).

When these findings are integrated with the *in vitro* adhesion assays, *L. reuteri* exhibited the highest adhesion ability among the five strains tested. Notably, *L. reuteri* SGL30004 possessed an expanded *lytG* gene that functions at the most downstream stage of the adhesion pathway. These results suggest that, rather than genes involved in precursor biosynthesis or surface protein display, genes directly involved in the final adhesion-promoting processes may exert the strongest influence on the adhesion ability of LAB.

**FIGURE 7:**
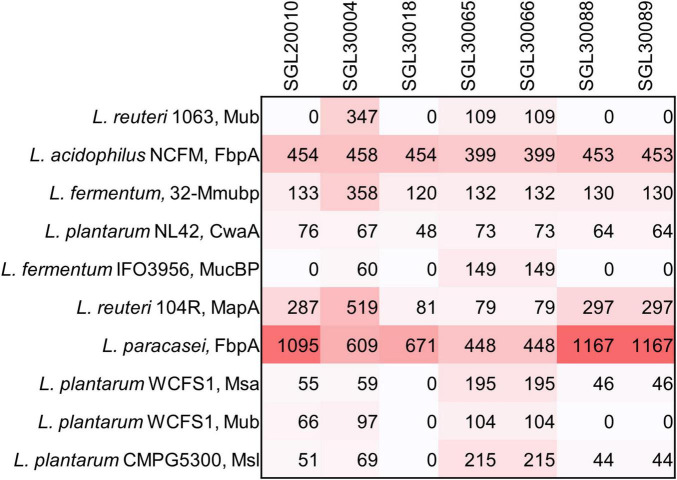
Distribution of adhesion-related proteins in genomes of lactic acid bacteria (LAB). Heatmap of the presence of known adhesion-associated proteins across the seven lactic acid bacterial strains analyzed in this study. Protein sequences reported in previous studies were used as reference queries, and homologous proteins were identified using BLASTp searches against each genome. Color intensity represents the bit score value of the top BLASTp alignment, indicating the relative similarity between the query and target proteins.

Recently, several cell surface proteins implicated in bacterial adhesion have been characterized, enabling the compilation of a list of known adhesion-associated proteins in LAB (Muscariello et al., 2020; Surve et al., 2022). Therefore, based on these previous reports, adhesion-related protein sequences were collected from the NCBI database and used as reference proteins for comparative analysis. The presence of these proteins or the associated homologs across the seven LAB strains in this study was evaluated using the BV-BRC BLASTp tool, and a heatmap displaying the bit score values of the top BLASTp hits was generated (Figure 7). Among the proteins examined, *L. reuteri* SGL30004 exhibited a higher number of identifiable adhesion-related proteins, suggesting that this strain may possess an enhanced capacity to adhere to host gut epithelial cells. However, the analysis was notably limited to a selected set of adhesion proteins, and other strains may harbor additional adhesion factors not captured in this dataset. Further studies assessing binding affinity to various gut cell types would provide deeper insights into strain-specific adhesion potential and clarify how these genomic features correspond to *in vitro* phenotypes.

#### 3.4.3 Secondary metabolite with antimicrobial activity

**FIGURE 8:**
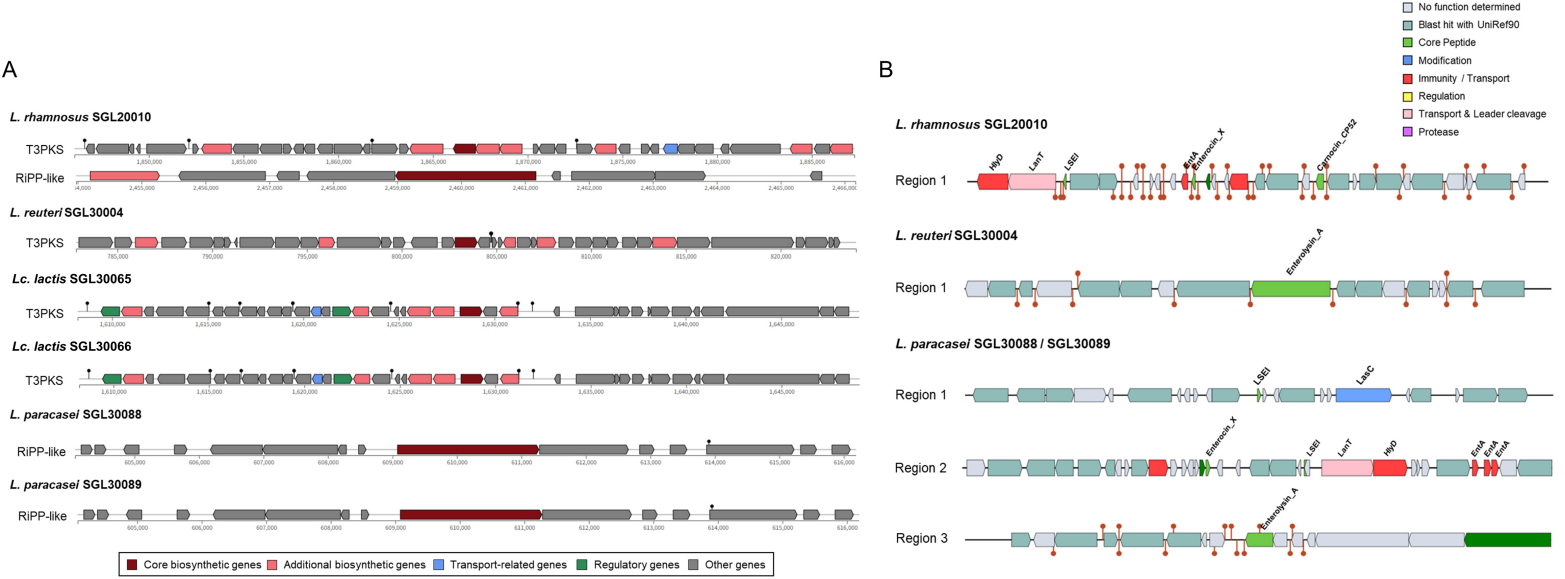
Prediction of antimicrobial biosynthetic gene clusters in genomes of LAB. **(A)** Secondary metabolite biosynthetic gene clusters predicted using the antiSMASH database. Identified clusters include Type III polyketide synthase (T3PKS) and ribosomally synthesized and post-translationally modified peptide (RiPP)-like clusters. **(B)** Bacteriocin biosynthetic gene clusters predicted using the BAGEL4 database. Identified clusters include genes encoding antimicrobial peptides such as enterocin_X, enterolysin_A, and LSEI family proteins associated with bacteriocin production.

According to the antiSMASH database, biosynthetic gene clusters were detected in six lactic acid bacterial strains, except for *L. curvatus* SGL30018. Unlike the other strains, which each possessed a single cluster, *L. rhamnosus* SGL20010, identified as having the strongest antibacterial activity in the *in vitro* characterization assay, contained two clusters: one Type III polyketide synthase (T3PKS) cluster and one ribosomally synthesized and post-translationally modified peptide (RiPP)–like cluster. T3PKS gene clusters are known to be involved in the biosynthesis of diverse polyketide compounds, many of which exhibit antimicrobial activity through disruption of microbial cell membranes or inhibition of essential metabolic pathways (Shimizu et al., 2017; Cai and Zhang, 2018). In addition, RiPP–like clusters are commonly associated with the production of bacteriocins and related antimicrobial peptides in lactic acid bacteria, which exert antibacterial effects by targeting membrane integrity or intracellular processes (Arnison et al., 2013). Therefore, the presence of both T3PKS and RiPP-like clusters in *L. rhamnosus* SGL20010 may contribute to its enhanced antibacterial activity observed *in vitro*. In contrast, *L. reuteri* SGL30004, *Lc. lactis* SGL30065, and SGL30066 each carried one T3PKS cluster, while *L. paracasei* SGL30088 and SGL30089 each harbored one RiPP-like cluster (Figure 8A). Across all strains in which T3PKS clusters were identified, the core biosynthetic gene was *mvaS*, which encodes hydroxymethylglutaryl-CoA synthase. Similarly, all RiPP-like clusters detected in these strains contained *lagD*, which encodes the ATP-binding protein involved in lactococcin-G processing and transport, as the core gene. An annotation of each gene in the clusters is provided in Supplementary Table 8.

Bacteriocin gene cluster prediction using the BAGEL4 database further indicated the presence of clusters in *L. rhamnosus* SGL20010, *L. reuteri* SGL30004, and *L. paracasei* SGL30088 and SGL30089 (Figure 8B). In SGL20010, a single cluster was identified, containing enterocin_X, carnocin_CP52, and LSEI_2386 as core genes. SGL30004 harbored a single cluster with enterolysin_A as the core gene. In both SGL30088 and SGL30089, three clusters were predicted: the first containing LSEI_2163 as the core gene, the second containing enterocin_X together with LSEI_2386, and the third containing enterolysin_A as the core gene. Previous studies have concluded that LSEI_2386 and LSEI_2163 correspond to genes encoding ribosomally synthesized antimicrobial peptides produced by LAB (Kuo et al., 2013).

## 4 Discussion

This study revealed substantial strain-specific variation in key probiotic-related traits, including acid tolerance, adhesion capacity, antimicrobial activity, and antibiotic susceptibility, highlighting the importance of evaluating LAB at the strain level rather than relying solely on species classification. These findings indicate that phenotypic diversity among LAB strains cannot be fully explained by species identity alone and underscore the need for integrated approaches to better understand the genetic basis underlying these traits. It should also be noted that the *in vitro* conditions used in this study represent simplified screening models, and further studies using more physiologically relevant and dynamic systems will be required to validate these findings. In particular, the antimicrobial activity of LAB-derived cell-free culture supernatants (CFCS) may arise from multiple factors, including organic acids, hydrogen peroxide, and bacteriocin-like peptides (Wang and Zeng, 2022). Without specific treatments such as pH neutralization or enzyme assays, it is difficult to distinguish the individual contributions of these factors. Accordingly, further studies are needed to identify and characterize the specific antimicrobial compounds involved. In addition, antibiotic resistance in LAB should be carefully interpreted in the context of probiotic safety. Resistance to certain antibiotics, such as vancomycin and aminoglycosides, is often considered intrinsic and is generally not associated with horizontal gene transfer. However, acquired resistance mediated by mobile genetic elements may pose a safety concern due to the potential for horizontal transfer to pathogenic bacteria. Therefore, further genomic analysis is required to distinguish between intrinsic and acquired resistance mechanisms and to evaluate the potential transferability of resistance genes.

Whole-genome sequencing and comparative genomics provided further insight into the genetic basis of these phenotypes, particularly regarding stress responses, amino acid metabolism, cell adhesion mechanisms, and secondary metabolite biosynthesis. Analysis of acid tolerance revealed that the distribution of general stress response genes did not differ substantially between tolerant and non-tolerant strains, suggesting that classical chaperone- and proton pump-mediated stress responses alone do not fully account for the observed phenotypic differences. Instead, genes involved in amino acid metabolism, including pathways associated with proton consumption or cell wall reinforcement, appeared to correlate more strongly with acid tolerance patterns. Notably, *L. curvatus* SGL30018 possessed markedly fewer genes involved in the conversion of L-aspartate to meso-DAP than the other strains, supporting the view that amino acid metabolic pathways contribute more prominently to acid tolerance in LAB than previously assumed.

Comparative analysis of adhesion-related genes identified expanded gene families that may underlie variation in adhesion ability across strains, including *galE*, *lgt*, *ftsW*, *ypeA*, and *lytG*. These genes participate in distinct stages of the adhesion pathway, from biosynthesis and structural modification of the cell envelope to lipoprotein maturation and autolysin-mediated remodeling. Among these, *lytG*, which was expanded exclusively in *L. reuteri* SGL30004, may be associated with the strong adhesion phenotype observed in this strain by altering surface charge, hydrophobicity, and eDNA release through cell wall hydrolysis.

Genomic analysis of antimicrobial-related pathways also revealed substantial species-level differences. Predictions from antiSMASH and BAGEL4 identified T3PKS clusters and RiPP/bacteriocin-associated clusters in multiple strains, with *L. rhamnosus* SGL20010 uniquely harboring both a T3PKS cluster and a RiPP-like cluster, consistent with known strong *in vitro* antimicrobial activity. Several core genes, including *mvaS*, *lagD*, *enterolysin_A*, LSEI_2386, and LSEI_2163, were detected; notably, LSEI_2386 and LSEI_2163 have previously been characterized as ribosomally synthesized antimicrobial peptides in LAB. These findings suggest that species-level differences in secondary metabolite biosynthesis may be associated with the observed variation in antimicrobial activity among the isolates.

Although species-level genomic differences were evident, strain-level differences were far subtler. In several cases, whole-genome comparisons alone were insufficient to explain the distinct phenotypes observed among strains within the same species. For example, *Lc. lactis* SGL30066 and *L. paracasei* SGL30088 exhibited unique phenotypic characteristics, such as acid tolerance and adhesion, respectively, that were not readily explained by genomic content alone. This suggests that regulatory differences, such as variation in gene expression rather than gene presence or absence, may underlie these strain-specific phenotypes. Therefore, additional experiments, such as transcriptomic profiling under relevant environmental conditions, will be necessary to determine whether differential expression of key genes contributes to these phenotypic disparities.

Overall, by integrating phenotypic and genomic analyses, this study highlights several candidate genes that may influence probiotic properties in LAB. Future functional studies should validate the roles of these genes through targeted genetic approaches. In particular, knockout experiments targeting adhesion-associated and antimicrobial-related genes would allow a direct assessment of the associated contributions to acid tolerance, adhesion, antimicrobial activity, and other key traits. Such functional analyses, together with transcriptomic investigations of strains exhibiting unexplained phenotypic differences, will provide deeper insight into the molecular mechanisms governing probiotic efficacy in LAB. It should be noted that the associations between genomic features and phenotypic traits identified in this study are based on correlative analyses, and further functional validation, such as gene knockout or expression studies, will be required to establish causal relationships.

## Data Availability

The assembled genome and annotation results have been deposited in the figshare database and can be accessed at https://doi.org/10.6084/m9.figshare.32602653.

## Footnotes

1
https://blast.ncbi.nlm.nih.gov/Blast.cgi
