# Supplementary material for: Functional and genomic profiling of lactic acid bacteria reveals specific traits as potential probiotics
Source: Front Microbiol. 2026 Jun 22;17:1850174. doi: 10.3389/fmicb.2026.1850174 (PMC13333773; doi:10.3389/fmicb.2026.1850174)
Supplement: Supplementary file 1 [file Data_Sheet_1.docx]

***Supplementary material***

**Table S1.** Interpretive criteria for inhibition zone diameters used to determine antibiotic susceptibility and resistance by the disk diffusion method.

| **Antibiotic name** | **Concentration** | **Susceptibility category** | | |
| --- | --- | --- | --- | --- |
|  |  | **Susceptible** | **Moderately susceptible** | **Resistant** |
| Penicillin | 10units/ml | ≥ 28mm | 20-27mm | ≤ 19mm |
| Vancomycin | 3mg/ml | ≥ 12mm | 10-11mm | ≤ 9mm |
| Cephalothin | 3mg/ml | ≥ 18mm | 15-17mm | ≤ 14mm |
| Gentamicin | 1mg/ml | ≥ 15mm | 13-14mm | ≤ 12mm |
| Chloramphenicol | 3mg/ml | ≥ 18mm | 13-17mm | ≤ 12mm |
| Tetracycline | 3mg/ml | ≥ 15mm | 12-14mm | ≤ 11mm |

**Table S2.** Genome assemblies used for phylogenetic analysis

| **Species** | **NCBI Accession** |
| --- | --- |
| *Lactobacillus curvatus* DSM20019 | GCA_004101845.1 |
| *Lactobacillus rhamnosus* 1.0320 | GCA_006151905.1 |
| *Lactobacillus paracasei* 362.5013889 | GCA_000155515.2 |
| *Lactobacillus acidophilus* ATCC4356 | GCA_034298135.1 |
| *Lactobacillus intestinalis* DSM6629 | GCA_001435325.1 |
| *Lactobacillus xylocopicola* Kim32-2 | GCA_033096005.1 |
| *Lactobacillus johnsonii* NCK2677 | GCA_014058685.1 |
| *Lactobacillus gasseri* ATCC33323 | GCA_000014425.1 |
| *Lactobacillus paragasseri* JCM5343 | GCA_003584685.1 |
| *Lactobacillus terrae* NIBRBAC000499792 | GCA_002762335.1 |
| *Lactobacillus reuteri* 2010 | GCA_003703885.1 |
| *Lactococcus lactis* 14B4 | GCA_003176835.1 |
| *Escherichia coli* K12 | GCA_000005845.2 |
| *Bacillus subtilis* 168 | GCA_000009045.1 |
| *Lactobacillus rhamnosus* SGL20010  *Lactobacillus reuteri* SGL30004  *Lactobacillus curvatus* SGL30018  *Lactococcus lactis* SGL30065  *Lactococcus lactis* SGL30066  *Lactobacillus paracasei* SGL30088  *Lactobacillus paracasei* SGL30089 | This study |

**Table S3.** Lactic acid bacterial strains isolated from the oral cavity and cheese samples.

| **Source** | **Isolate name** | **Matches to 16S rRNA sequences from GenBank database** | **Identity** |
| --- | --- | --- | --- |
| Oral cavity | SGL20001 | *Lactobacillus rhamnosus* strain NBRC 3425 | 99.70% |
|  | SGL20002 | *Lactobacillus rhamnosus* strain NBRC 3425 | 99.70% |
|  | SGL20003 | *Lactobacillus rhamnosus* strain NBRC 3425 | 99.70% |
|  | SGL20004 | *Lactobacillus rhamnosus* strain NBRC 3425 | 99.70% |
|  | SGL20005 | *Lactobacillus rhamnosus* strain NBRC 3425 | 99.70% |
|  | SGL20006 | *Lactobacillus rhamnosus* strain NBRC 3425 | 99.70% |
|  | SGL20007 | *Lactobacillus rhamnosus* strain NBRC 3425 | 99.70% |
|  | SGL20008 | *Lactobacillus rhamnosus* strain NBRC 3425 | 99.70% |
|  | SGL20009 | *Lactobacillus rhamnosus* strain NBRC 3425 | 99.70% |
|  | SGL20010 | *Lactobacillus rhamnosus* strain NBRC 3425 | 99.70% |
|  | SGL20011 | *Lactobacillus rhamnosus* strain NBRC 3425 | 99.70% |
| Cheese | SGL30004 | *Lactobacillus reuteri* strain NBRC 15892 | 100.00% |
|  | SGL30005 | *Lactobacillus reuteri* strain NBRC 15892 | 100.00% |
|  | SGL30006 | *Lactobacillus reuteri* strain NBRC 15892 | 100.00% |
|  | SGL30007 | *Lactobacillus reuteri* strain NBRC 15892 | 100.00% |
|  | SGL30018 | *Lactobacillus curvatus* strain DSM 20019 | 99.88% |
|  | SGL30019 | *Lactobacillus curvatus* strain DSM 20019 | 99.37% |
|  | SGL30020 | *Lactobacillus curvatus* strain DSM 20019 | 99.02% |
|  | SGL30022 | *Lactobacillus curvatus* strain DSM 20019 | 99.87% |
|  | SGL30065 | *Lactococcus lactis* strain NCDO 604 | 100.00% |
|  | SGL30066 | *Lactococcus lactis* strain NCDO 604 | 100.00% |
|  | SGL30067 | *Lactococcus lactis* strain NCDO 604 | 100.00% |
|  | SGL30068 | *Lactococcus lactis* strain NCDO 604 | 100.00% |
|  | SGL30088 | *Lactobacillus paracasei CIRM-BIA2373* | 99.71% |
|  | SGL30089 | *Lactobacillus paracasei CIRM-BIA2373* | 99.71% |
|  | SGL30091 | *Lactobacillus paracasei CIRM-BIA2373* | 99.71% |
|  | SGL30092 | *Lactobacillus paracasei CIRM-BIA2373* | 99.71% |

**Table S4.** Acid and bile survival rates and adhesion ability of 27 tested lactic acid bacterial isolates.

| Isolate  name | Species | Survival rate (%)^*^ | | |
| --- | --- | --- | --- | --- |
|  |  | **pH 4.0 acid tolerance** | **0.3% bile tolerance** | **Adherent ability to HT-29 cell** |
| SGL20001 | *L. rhamnosus* | 85.0 ± 1.22^a^ | 94.80 ± 1.26^a^ | 49.18 ± 4.44^a^ |
| SGL20002 | *L. rhamnosus* | 88.94 ± 0.47^a^ | 87.96 ± 2.17^a^ | 49.70 ± 6.67^a^ |
| SGL20003 | *L. rhamnosus* | 91.00 ± 1.14^a^ | 87.57 ± 2.53^a^ | 50.28 ± 4.50^a^ |
| SGL20004 | *L. rhamnosus* | 99.45 ± 1.67^b^ | 90.49 ± 0.68^a^ | 48.57 ± 5.60^a^ |
| SGL20005 | *L. rhamnosus* | 94.74 ± 2.11^a^ | 83.40 ± 1.46^b^ | 48.91 ± 4.37^a^ |
| SGL20006 | *L. rhamnosus* | 102.03 ± 1.17^b^ | 85.59 ± 0.99^b^ | 49.89 ± 6.17^a^ |
| SGL20007 | *L. rhamnosus* | 103.02 ± 1.55^b^ | 50.66 ± 4.00^d^ | 50.02 ± 4.98^a^ |
| SGL20008 | *L. rhamnosus* | 98.18 ± 2.43^b^ | 64.58 ± 0.62^c^ | 49.85 ± 6.83^a^ |
| SGL20009 | *L. rhamnosus* | 105.62 ± 0.71^b^ | 81.69 ± 0.93^b^ | 49.77 ± 3.90^a^ |
| SGL20010 | *L. rhamnosus* | 109.64 ± 0.91^b^ | 107.76 ± 0.78^e^ | 46.68 ± 5.69^a^ |
| SGL20011 | *L. rhamnosus* | 107.93 ± 0.42^b^ | 90.46 ± 0.70^a^ | 48.79 ± 5.71^a^ |
| SGL30004 | *L. reuteri* | 107.73 ± 0.63^b^ | 101.76 ± 0.86^a^ | 78.42 ± 3.10^b^ |
| SGL30005 | *L. reuteri* | 100.19 ± 4.44^b^ | 95.24 ± 0.62^a^ | 79.48 ± 1.65^b^ |
| SGL30006 | *L. reuteri* | 93.14 ± 1.28^a^ | 104.28 ± 3.91^b^ | 83.22 ± 2.58^b^ |
| SGL30007 | *L. reuteri* | 91.82 ± 2.41^a^ | 99.79 ± 4.42^a^ | 81.18 ± 0.31^b^ |
| SGL30018 | *L. curvatus* | 0.00 ± 0.00 | - | 42.62 ± 6.16^c^ |
| SGL30019 | *L. curvatus* | 0.00 + 0.00 | - | 48.46 ± 0.57^c^ |
| SGL30020 | *L. curvatus* | 0.00 ± 0.00 | - | 63.59 ± 3.08^c^ |
| SGL30022 | *L. curvatus* | 0.00 ± 0.00 | - | 53.51 ± 2.02^c^ |
| SGL30065 | *Lc. lactis* | 0.00 ± 0.00 | - | 63.83 ± 2.49^c^ |
| SGL30066 | *Lc. lactis* | 83.06 ± 2.58^c^ | 69.70 ± 2.63^f^ | 0.00 ± 0.00 |
| SGL30067 | *Lc. lactis* | 0.00 ± 0.00 | - | 50.58 ± 4.17^c^ |
| SGL30068 | *Lc. lactis* | 0.00 ± 0.00 | - | 49.47 ± 4.87^c^ |
| SGL30088 | *L. paracasei* | 107.33 ± 1.07^d^ | 86.55 ± 1.53^b^ | 52.32 ± 2.91^c^ |
| SGL30089 | *L. paracasei* | 0.00 ± 0.00 | - | 70.65 ± 1.32^d^ |
| SGL30091 | *L. paracasei* | 0.00 ± 0.00 | - | 65.40 ± 3.51^c^ |
| SGL30092 | *L. paracasei* | 0.00 ± 0.00 | - | 67.64 ± 0.56^c^ |

*Data are expressed as the mean ± standard error of the mean; ^abcde^ indicate significant differences among groups in the same row (p < 0.05)

**Table S5.** Antibacterial activity of lactic acid bacterial isolates against *E. coli*, *S. aureus*, and *C. albicans*.

| **Isolate name** | **Species** | **Survival rate (%)**^*^ | | |
| --- | --- | --- | --- | --- |
|  |  | *E. coli* | *S. aureus* | *C. albicans* |
| SGL20001 | *L. rhamnosus* | 62.56 ± 1.75^a^ | 87.16 ± 2.03^a^ | 65.15 ± 3.18^a^ |
| SGL20002 | *L. rhamnosus* | 40.59 ± 0.95^b^ | 65.93 ± 0.37^b^ | 65.88 ± 0.77^a^ |
| SGL20003 | *L. rhamnosus* | 42.80 ± 6.25^b^ | 65.89 ± 3.88^b^ | 75.13 ± 2.06^ab^ |
| SGL20004 | *L. rhamnosus* | 43.50 ± 1.54^b^ | 77.90 ± 1.89^b^ | 66.07 ± 3.11^a^ |
| SGL20005 | *L. rhamnosus* | 45.73 ± 0.82^b^ | 46.94 ± 2.43^b^ | 63.60 ± 2.53^a^ |
| SGL20006 | *L. rhamnosus* | 44.90 ± 4.16^b^ | 31.91 ± 1.29^b^ | 71.61 ± 1.31^a^ |
| SGL20007 | *L. rhamnosus* | 43.87 ± 1.72^b^ | 35.38 ± 4.05^b^ | 71.56 ± 0.90^a^ |
| SGL20008 | *L. rhamnosus* | 44.03 ± 4.19^b^ | 38.22 ± 2.02^b^ | 75.69 ± 3.66^ab^ |
| SGL20009 | *L. rhamnosus* | 46.61 ± 2.51^b^ | 44.48 ± 4.25^b^ | 73.08 ± 1.79^a^ |
| SGL20010 | *L. rhamnosus* | 47.71 ± 0.80^b^ | 45.83 ± 1.44^b^ | 56.33 ± 1.96^h^ |
| SGL20011 | *L. rhamnosus* | 47.77 ± 1.63^b^ | 37.80 ± 2.20^b^ | 74.43 ± 1.98^a^ |
| SGL30004 | *L. reuteri* | 83.33 ± 2.09^c^ | 91.92 ± 1.57^ac^ | 121.65 ± 0.95^g^ |
| SGL30005 | *L. reuteri* | 104.44 ± 3.15^d^ | 94.65 ± 3.98^ac^ | 78.30 ± 2.24^bc^ |
| SGL30006 | *L. reuteri* | 100.31 ± 1.28^d^ | 97.30 ± 2.54^ac^ | 76.84 ± 2.87^bc^ |
| SGL30007 | *L. reuteri* | 105.60 ± 1.20^d^ | 101.38 ± 2.96^ac^ | 71.47 ± 1.03^bc^ |
| SGL30018 | *L. curvatus* | 104.76 ± 3.32^d^ | 93.02 ± 2.16^ac^ | 71.84 ± 1.44^bc^ |
| SGL30019 | *L. curvatus* | 108.13 ± 0.20^d^ | 86.90 ± 2.46^ac^ | 66.76 ± 1.79^bc^ |
| SGL30020 | *L. curvatus* | 103.51 ± 5.88^d^ | 95.41 ± 1.00^ac^ | 75.88 ± 4.00^bc^ |
| SGL30022 | *L. curvatus* | 104.34 ± 2.77^d^ | 93.38 ± 2.01^ac^ | 86.09 ± 0.44^cd^ |
| SGL30065 | *Lc. lactis* | 99.00 ± 9.82^d^ | 91.93 ± 1.17^ac^ | 104.59 ± 3.04^d^ |
| SGL30066 | *Lc. lactis* | 154.62 ± 0.80^g^ | 135.23 ± 4.05^f^ | 104.34 ± 2.49^d^ |
| SGL30067 | *Lc. lactis* | 141.73 ± 0.82^e^ | 105.30 ± 3.51^d^ | 88.61 ± 6.19^e^ |
| SGL30068 | *Lc. lactis* | 96.78 ± 1.95^d^ | 99.58 ± 0.83^ac^ | 86.73 ± 1.87^e^ |
| SGL30088 | *L. paracasei* | 103.21 ± 1.63^d^ | 93.53 ± 4.50^ac^ | 83.59 ± 2.48^e^ |
| SGL30089 | *L. paracasei* | 118.50 ± 2.03^f^ | 83.74 ± 1.29^cd^ | 95.85 ± 1.68^f^ |
| SGL30091 | *L. paracasei* | 106.75 ± 2.28^d^ | 82.42 ± 1.70^cd^ | 96.20 ± 1.22^f^ |
| SGL30092 | *L. paracasei* | 113.04 ± 2.49^d^ | 122.21 ± 2.34^e^ | 96.90 ± 3.05^f^ |

* Data are expressed as the mean ± standard error of the mean; ^abcdefgh^ indicate significant differences among groups in the same row (p < 0.05)

**Table S6.** Antibiotic susceptibility test of lactic acid bacterial isolates.

| Isolate name | Species | P | V | CF | TE | C | GM |
| --- | --- | --- | --- | --- | --- | --- | --- |
| SGL20001 | *L. rhamnosus* | R | R | R | S | S | R |
| SGL20002 | *L. rhamnosus* | R | R | MS | S | S | R |
| SGL20003 | *L. rhamnosus* | R | R | R | S | S | R |
| SGL20004 | *L. rhamnosus* | R | R | S | S | S | R |
| SGL20005 | *L. rhamnosus* | R | R | R | S | S | R |
| SGL20006 | *L. rhamnosus* | R | R | MS | S | S | R |
| SGL20007 | *L. rhamnosus* | R | R | S | S | S | R |
| SGL20008 | *L. rhamnosus* | R | R | R | S | S | R |
| SGL20009 | *L. rhamnosus* | R | R | R | S | S | R |
| SGL20010 | *L. rhamnosus* | R | R | R | S | S | R |
| SGL20011 | *L. rhamnosus* | R | R | MS | S | S | R |
| SGL30004 | *L. reuteri* | R | R | S | MS | S | R |
| SGL30005 | *L. reuteri* | R | R | S | R | S | R |
| SGL30006 | *L. reuteri* | R | R | S | S | S | R |
| SGL30007 | *L. reuteri* | R | R | S | MS | S | R |
| SGL30018 | *L. curvatus* | R | R | S | S | S | R |
| SGL30019 | *L. curvatus* | R | R | S | S | S | R |
| SGL30020 | *L. curvatus* | R | R | S | S | S | R |
| SGL30022 | *L. curvatus* | R | R | S | S | S | R |
| SGL30065 | *Lc. lactis* | R | S | S | S | S | R |
| SGL30066 | *Lc. lactis* | R | S | S | S | S | R |
| SGL30067 | *Lc. lactis* | MS | S | S | R | R | R |
| SGL30068 | *Lc. lactis* | R | S | S | S | S | R |
| SGL30088 | *L. paracasei* | R | R | S | S | S | R |
| SGL30089 | *L. paracasei* | R | R | S | S | S | R |
| SGL30091 | *L. paracasei* | R | R | MS | R | R | R |
| SGL30092 | *L. paracasei* | R | R | R | R | S | R |

S: susceptible, MS : moderately susceptible, R: resistant

P: Penicillin (10 units), V: Vancomycin (30 μg), CF: Cephalothin (30 μg), TE: Tetracycline (30 μg), C: Chloramphenicol (30 μg), GM: Gentamicin (10 μg)

| **Gene** | **Transcription** | **SGL20010** | **SGL30004** | **SGL30018** | **SGL30065** | **SGL30066** | **SGL30088** | **SGL30089** |
| --- | --- | --- | --- | --- | --- | --- | --- | --- |
| *pepN* | Aminopeptidase N | CJBMIEPA_00550 | JEGPBKMD_02053 | GEBIHPBK_01849 | LNEACJHF_00372 | OIAAFOCL_00372 | DOPMHDDG_02633 | AIKLKJDG_02633 |
| *pepC* | Aminopeptidase C | CJBMIEPA_02299 | JEGPBKMD_00350 | GEBIHPBK_01750 | LNEACJHF_02124 | OIAAFOCL_02124 | DOPMHDDG_00663 | AIKLKJDG_00663 |
| *pepE* | Aminopeptidase E | CJBMIEPA_02298 | JEGPBKMD_02050 | GEBIHPBK_01749 | #N/A | #N/A | DOPMHDDG_00664 | AIKLKJDG_00664 |
| *pepT* | Peptidase T | CJBMIEPA_00349 | JEGPBKMD_00859 | GEBIHPBK_01021 | LNEACJHF_02044 | OIAAFOCL_02044 | #N/A | #N/A |
| *pepV* | Beta-Ala-Xaa dipeptidase | #N/A | #N/A | GEBIHPBK_01678 | LNEACJHF_00924 | OIAAFOCL_00924 | DOPMHDDG_02355 | AIKLKJDG_02355 |
| *artP* | Arginine-binding extracellular protein | #N/A | #N/A | GEBIHPBK_00992 | LNEACJHF_00640 | OIAAFOCL_00640 | DOPMHDDG_01720 | AIKLKJDG_01720 |
| *artQ* | Arginine transport system permease protein | CJBMIEPA_01257 | JEGPBKMD_00605 | GEBIHPBK_00990 | LNEACJHF_02030 | OIAAFOCL_02030 | DOPMHDDG_01722 | AIKLKJDG_01722 |
| *artM* | Arginine transport ATP-binding protein | CJBMIEPA_02791 | JEGPBKMD_00838 | GEBIHPBK_00991 | #N/A | #N/A | DOPMHDDG_02763 | AIKLKJDG_02763 |
| *gltT* | Proton/sodium-glutamate symport protein | #N/A | JEGPBKMD_01930 | GEBIHPBK_01617 | #N/A | #N/A | #N/A | #N/A |
| *yveA* | Aspartate-proton symporter | CJBMIEPA_00826 | JEGPBKMD_00412 | GEBIHPBK_00638 | #N/A | #N/A | DOPMHDDG_02305 | AIKLKJDG_02305 |

**Table S7.** Presence of aminopeptidase genes and amino acid transport-related genes identified in the genomes of lactic acid bacteria (LAB).

**Table S8.** Gene clusters encoding secondary metabolic enzymes in the genomes of lactic acid bacterial strains.

| Strain | Cluster | Gene ID | From | To | Size (nt) | Strand | Annotation |
| --- | --- | --- | --- | --- | --- | --- | --- |
| SGL20010 | T3PKS | CJBMIEPA_01798 | 1,846,682 | 1,847,113 | 432 | - | Hypothetical protein |
|  |  | CJBMIEPA_01799 | 1,847,206 | 1,848,930 | 1,725 | - | Phosphoenolpyruvate-protein phosphotransferase |
|  |  | CJBMIEPA_01800 | 1,848,930 | 1,849,196 | 267 | - | Phosphocarrier protein HPr |
|  |  | CJBMIEPA_01801 | 1,849,341 | 1,849,532 | 192 | - | Hypothetical protein |
|  |  | CJBMIEPA_01802 | 1,849,881 | 1,851,974 | 2,094 | + | ATP-dependent Clp protease ATP-binding subunit ClpE |
|  |  | CJBMIEPA_01803 | 1,852,321 | 1,852,614 | 294 | + | Hypothetical protein |
|  |  | CJBMIEPA_01804 | 1,852,782 | 1,854,356 | 1,575 | - | Peptide chain release factor 3 |
|  |  | CJBMIEPA_01805 | 1,854,424 | 1,855,770 | 1,347 | - | Hypothetical protein |
|  |  | CJBMIEPA_01806 | 1,855,845 | 1,857,023 | 1,179 | - | Hypothetical protein |
|  |  | CJBMIEPA_01807 | 1,857,100 | 1,857,534 | 435 | + | Acetyltransferase |
|  |  | CJBMIEPA_01808 | 1,857,642 | 1,858,160 | 519 | - | Hypothetical protein |
|  |  | CJBMIEPA_01809 | 1,858,184 | 1,858,753 | 570 | - | Hypothetical protein |
|  |  | CJBMIEPA_01810 | 1,858,901 | 1,859,698 | 798 | - | Regulatory protein RecX |
|  |  | CJBMIEPA_01811 | 1,859,770 | 1,861,290 | 1,521 | + | Hypothetical protein |
|  |  | CJBMIEPA_01812 | 1,861,297 | 1,861,677 | 381 | - | Hypothetical protein |
|  |  | CJBMIEPA_01813 | 1,861,755 | 1,863,122 | 1,368 | + | putative RNA methyltransferase |
|  |  | CJBMIEPA_01814 | 1,863,233 | 1,863,712 | 480 | + | Hypothetical protein |
|  |  | CJBMIEPA_01815 | 1,863,774 | 1,865,528 | 1,755 | - | Pyruvate oxidase |
|  |  | **CJBMIEPA_01816** | **1,866,069** | **1,867,241** | **1,173** | **-** | **Hydroxymethylglutaryl-CoA synthase** |
|  |  | CJBMIEPA_01817 | 1,867,246 | 1,868,505 | 1,260 | - | 3-hydroxy-3-methylglutaryl-coenzyme A reductase |
|  |  | CJBMIEPA_01818 | 1,868,518 | 1,869,699 | 1,182 | - | Acetyl-CoA acetyltransferase |
|  |  | CJBMIEPA_01819 | 1,870,294 | 1,870,593 | 300 | + | Hypothetical protein |
|  |  | CJBMIEPA_01820 | 1,870,590 | 1,871,420 | 831 | - | Hypothetical protein |
|  |  | CJBMIEPA_01821 | 1,871,454 | 1,872,017 | 564 | - | Hypothetical protein |
|  |  | CJBMIEPA_01822 | 1,872,591 | 1,873,424 | 834 | + | Hypothetical protein |
|  |  | CJBMIEPA_01823 | 1,873,521 | 1,874,663 | 1,143 | - | putative succinyl-diaminopimelate desuccinylase |
|  |  | CJBMIEPA_01824 | 1,874,795 | 1,875,400 | 606 | - | Pantothenic acid transporter PanT |
|  |  | CJBMIEPA_01826 | 1,875,996 | 1,876,460 | 465 | + | Hypothetical protein |
|  |  | CJBMIEPA_01827 | 1,876,457 | 1,876,909 | 453 | - | Protein SprT-like protein |
|  |  | CJBMIEPA_01828 | 1,877,157 | 1,877,909 | 753 | - | Thiamine import ATP-binding protein ThiQ |
|  |  | CJBMIEPA_01829 | 1,877,902 | 1,878,801 | 900 | - | Hypothetical protein |
|  |  | CJBMIEPA_01830 | 1,878,798 | 1,879,793 | 996 | - | Hypothetical protein |
|  |  | CJBMIEPA_01831 | 1,880,178 | 1,880,615 | 438 | - | Hypothetical protein |
|  |  | CJBMIEPA_01832 | 1,880,673 | 1,883,336 | 2,664 | - | Calcium-transporting ATPase 1 |
|  |  | CJBMIEPA_01833 | 1,883,850 | 1,885,010 | 1,161 | - | Putative penicillin-binding protein PbpX |
|  |  | CJBMIEPA_01834 | 1,885,122 | 1,885,949 | 828 | - | NH(3)-dependent NAD(+) synthetase |
|  |  | CJBMIEPA_01835 | 1,885,949 | 1,887,145 | 1,197 | - | D-aminopeptidase |
|  | RIPP-like | CJBMIEPA_02333 | 2,454,197 | 2,455,279 | 1,083 | + | Aldo-keto reductase IolS |
|  |  | CJBMIEPA_02334 | 2,455,582 | 2,456,937 | 1,356 | - | Hypothetical protein |
|  |  | CJBMIEPA_02335 | 2,457,114 | 2,457,467 | 354 | - | Hypothetical protein |
|  |  | CJBMIEPA_02336 | 2,457,585 | 2,458,964 | 1,380 | - | Lactococcin A secretion protein LcnD |
|  |  | **CJBMIEPA_02337** | **2,458,977** | **2,461,169** | **2,193** | **-** | **Lactococcin-G-processing and transport ATP-binding protein LagD** |
|  |  | CJBMIEPA_02338 | 2,461,418 | 2,461,552 | 135 | - | Hypothetical protein |
|  |  | CJBMIEPA_02339 | 2,461,728 | 2,463,041 | 1,314 | + | Hypothetical protein |
|  |  | CJBMIEPA_02340 | 2,463,034 | 2,463,822 | 789 | + | Accessory gene regulator protein A |
|  |  | CJBMIEPA_02341 | 2,465,461 | 2,465,643 | 183 | - | Hypothetical protein |
| SGL30004 | T3PKS | JEGPBKMD_00777 | 782,964 | 784,745 | 1,782 | + | Ribonuclease J 2 |
|  |  | JEGPBKMD_00778 | 784,862 | 785,767 | 906 | + | Hypothetical protein |
|  |  | JEGPBKMD_00779 | 785,946 | 787,136 | 1,191 | + | Elongation factor Tu |
|  |  | JEGPBKMD_00780 | 787,332 | 788,642 | 1,311 | + | Trigger factor |
|  |  | JEGPBKMD_00781 | 788,820 | 790,070 | 1,251 | + | ATP-dependent Clp protease ATP-binding subunit ClpX |
|  |  | JEGPBKMD_00782 | 790,088 | 790,678 | 591 | + | putative GTP-binding protein EngB |
|  |  | JEGPBKMD_00783 | 790,638 | 790,979 | 342 | + | Hypothetical protein |
|  |  | JEGPBKMD_00784 | 791,177 | 791,314 | 138 | - | Hypothetical protein |
|  |  | JEGPBKMD_00785 | 791,457 | 793,268 | 1,812 | + | UvrABC system protein C |
|  |  | JEGPBKMD_00786 | 793,333 | 794,649 | 1,317 | + | GTPase Obg |
|  |  | JEGPBKMD_00787 | 794,682 | 795,611 | 930 | + | Ribonuclease Z |
|  |  | JEGPBKMD_00788 | 795,628 | 796,461 | 834 | + | Hypothetical protein |
|  |  | JEGPBKMD_00789 | 796,594 | 798,921 | 2,328 | + | Hypothetical protein |
|  |  | JEGPBKMD_00790 | 798,938 | 799,456 | 519 | + | Adenine phosphoribosyltransferase |
|  |  | JEGPBKMD_00791 | 799,567 | 800,181 | 615 | + | Hypothetical protein |
|  |  | JEGPBKMD_00793 | 800,497 | 801,879 | 1,383 | - | ISLre2 family transposase ISLre2 |
|  |  | JEGPBKMD_00794 | 802,127 | 802,795 | 669 | + | Ribonuclease H |
|  |  | **JEGPBKMD_00795** | **802,821** | **803,978** | **1,158** | **+** | **Hydroxymethylglutaryl-CoA synthase** |
|  |  | JEGPBKMD_00796 | 804,019 | 804,645 | 627 | - | LexA repressor |
|  |  | JEGPBKMD_00797 | 804,774 | 805,025 | 252 | + | Hypothetical protein |
|  |  | JEGPBKMD_00798 | 805,099 | 805,326 | 228 | + | Hypothetical protein |
|  |  | JEGPBKMD_00799 | 805,370 | 806,008 | 639 | - | 1-acyl-sn-glycerol-3-phosphate acyltransferase |
|  |  | JEGPBKMD_00800 | 806,103 | 806,858 | 756 | + | tRNA1(Val) (adenine(37)-N6)-methyltransferase |
|  |  | JEGPBKMD_00801 | 806,848 | 807,138 | 291 | + | Hypothetical protein |
|  |  | JEGPBKMD_00802 | 807,140 | 808,132 | 993 | + | D-lactate dehydrogenase |
|  |  | JEGPBKMD_00803 | 808,296 | 809,084 | 789 | + | 30S ribosomal protein S2 |
|  |  | JEGPBKMD_00804 | 809,182 | 810,057 | 876 | + | Elongation factor Ts |
|  |  | JEGPBKMD_00805 | 810,091 | 810,861 | 771 | + | Uridylate kinase |
|  |  | JEGPBKMD_00806 | 810,866 | 811,429 | 564 | + | Ribosome-recycling factor |
|  |  | JEGPBKMD_00807 | 811,653 | 812,417 | 765 | + | Ditrans,polycis-undecaprenyl-diphosphate synthase ((2E,6E)-farnesyl-diphosphate specific) |
|  |  | JEGPBKMD_00808 | 812,437 | 813,222 | 786 | + | Hypothetical protein |
|  |  | JEGPBKMD_00809 | 813,245 | 814,519 | 1,275 | + | Regulator of sigma-W protease RasP |
|  |  | JEGPBKMD_00810 | 814,545 | 816,278 | 1,734 | + | Proline--tRNA ligase |
|  |  | JEGPBKMD_00811 | 816,354 | 820,685 | 4,332 | + | DNA polymerase III PolC-type |
|  |  | JEGPBKMD_00812 | 820,819 | 821,292 | 474 | + | Ribosome maturation factor RimP |
|  |  | JEGPBKMD_00813 | 821,313 | 822,500 | 1,188 | + | Transcription termination/antitermination protein NusA |
|  |  | JEGPBKMD_00814 | 822,550 | 822,831 | 282 | + | Hypothetical protein |
|  |  | JEGPBKMD_00815 | 822,818 | 823,129 | 312 | + | putative ribosomal protein YlxQ |
| SGL30065 | T3PKS | LNEACJHF_01638 | 1,609,413 | 1,610,387 | 975 | - | HTH-type transcriptional regulator HdfR |
|  |  | LNEACJHF_01639 | 1,610,485 | 1,611,561 | 1,077 | - | Hydroxycarboxylate dehydrogenase A |
|  |  | LNEACJHF_01640 | 1,611,664 | 1,612,158 | 495 | - | Hypothetical protein |
|  |  | LNEACJHF_01641 | 1,612,297 | 1,613,721 | 1,425 | - | Dipeptidase A |
|  |  | LNEACJHF_01642 | 1,613,837 | 1,615,072 | 1,236 | - | CCA-adding enzyme |
|  |  | LNEACJHF_01643 | 1,615,253 | 1,615,789 | 537 | - | Hypothetical protein |
|  |  | LNEACJHF_01644 | 1,615,838 | 1,616,620 | 783 | - | 4-hydroxy-tetrahydrodipicolinate reductase |
|  |  | LNEACJHF_01645 | 1,616,721 | 1,617,566 | 846 | - | DegV domain-containing protein |
|  |  | LNEACJHF_01646 | 1,617,584 | 1,617,976 | 393 | - | Hypothetical protein |
|  |  | LNEACJHF_01647 | 1,618,102 | 1,618,857 | 756 | - | tRNA (guanine-N(1)-)-methyltransferase |
|  |  | LNEACJHF_01648 | 1,618,850 | 1,619,365 | 516 | - | Ribosome maturation factor RimM |
|  |  | LNEACJHF_01649 | 1,619,517 | 1,620,329 | 813 | - | Pyrroline-5-carboxylate reductase |
|  |  | LNEACJHF_01650 | 1,620,401 | 1,620,928 | 528 | - | Hypothetical protein |
|  |  | LNEACJHF_01651 | 1,620,919 | 1,621,395 | 477 | - | Hypothetical protein |
|  |  | LNEACJHF_01652 | 1,621,538 | 1,622,497 | 960 | + | HTH-type transcriptional activator RhaR |
|  |  | LNEACJHF_01653 | 1,622,562 | 1,623,416 | 855 | - | Phospholipase YtpA |
|  |  | LNEACJHF_01654 | 1,623,530 | 1,624,474 | 945 | - | Ferrochelatase |
|  |  | LNEACJHF_01655 | 1,624,736 | 1,624,975 | 240 | - | Hypothetical protein |
|  |  | LNEACJHF_01656 | 1,625,071 | 1,625,343 | 273 | - | 30S ribosomal protein S16 |
|  |  | LNEACJHF_01657 | 1,625,432 | 1,626,643 | 1,212 | - | 3-hydroxy-3-methylglutaryl-coenzyme A reductase |
|  |  | LNEACJHF_01658 | 1,626,745 | 1,627,884 | 1,140 | - | Acetyl-CoA acetyltransferase |
|  |  | **LNEACJHF_01659** | **1,628,193** | **1,629,347** | **1,155** | **+** | **Hydroxymethylglutaryl-CoA synthase** |
|  |  | LNEACJHF_01660 | 1,629,407 | 1,630,114 | 708 | - | Glucosamine-6-phosphate deaminase |
|  |  | LNEACJHF_01661 | 1,630,265 | 1,631,224 | 960 | - | 2-succinyl-6-hydroxy-2,4-cyclohexadiene-1-carboxylate synthase |
|  |  | LNEACJHF_01662 | 1,633,093 | 1,633,389 | 297 | - | Hypothetical protein |
|  |  | LNEACJHF_01663 | 1,634,226 | 1,636,286 | 2,061 | + | Chromosome partition protein Smc |
|  |  | LNEACJHF_01664 | 1,636,252 | 1,636,515 | 264 | + | Hypothetical protein |
|  |  | LNEACJHF_01665 | 1,636,512 | 1,637,063 | 552 | + | Hypothetical protein |
|  |  | LNEACJHF_01666 | 1,637,182 | 1,637,772 | 591 | + | Replication protein RepB |
|  |  | LNEACJHF_01667 | 1,638,033 | 1,638,188 | 156 | + | Hypothetical protein |
|  |  | LNEACJHF_01668 | 1,638,185 | 1,639,150 | 966 | + | Tyrosine recombinase XerC |
|  |  | LNEACJHF_01669 | 1,639,294 | 1,640,331 | 1,038 | + | S-adenosylmethionine:tRNA ribosyltransferase-isomerase |
|  |  | LNEACJHF_01670 | 1,640,407 | 1,640,718 | 312 | - | Hypothetical protein |
|  |  | LNEACJHF_01671 | 1,640,797 | 1,641,969 | 1,173 | - | Hypothetical protein |
|  |  | LNEACJHF_01672 | 1,642,092 | 1,646,948 | 4,857 | - | Hypothetical protein |
|  |  | LNEACJHF_01673 | 1,646,985 | 1,647,740 | 756 | - | Hypothetical protein |
|  |  | LNEACJHF_01674 | 1,647,778 | 1,648,536 | 759 | - | Hypothetical protein |
| SGL30066 | T3PKS | OIAAFOCL_01638 | 1,609,420 | 1,610,394 | 975 | - | HTH-type transcriptional regulator HdfR |
|  |  | OIAAFOCL_01639 | 1,610,492 | 1,611,568 | 1,077 | - | Hydroxycarboxylate dehydrogenase A |
|  |  | OIAAFOCL_01640 | 1,611,671 | 1,612,165 | 495 | - | Hypothetical protein |
|  |  | OIAAFOCL_01641 | 1,612,304 | 1,613,728 | 1,425 | - | Dipeptidase A |
|  |  | OIAAFOCL_01642 | 1,613,844 | 1,615,079 | 1,236 | - | CCA-adding enzyme |
|  |  | OIAAFOCL_01643 | 1,615,260 | 1,615,796 | 537 | - | Hypothetical protein |
|  |  | OIAAFOCL_01644 | 1,615,845 | 1,616,627 | 783 | - | 4-hydroxy-tetrahydrodipicolinate reductase |
|  |  | OIAAFOCL_01645 | 1,616,728 | 1,617,573 | 846 | - | DegV domain-containing protein |
|  |  | OIAAFOCL_01646 | 1,617,591 | 1,617,983 | 393 | - | Hypothetical protein |
|  |  | OIAAFOCL_01647 | 1,618,109 | 1,618,864 | 756 | - | tRNA (guanine-N(1)-)-methyltransferase |
|  |  | OIAAFOCL_01648 | 1,618,857 | 1,619,372 | 516 | - | Ribosome maturation factor RimM |
|  |  | OIAAFOCL_01649 | 1,619,524 | 1,620,336 | 813 | - | Pyrroline-5-carboxylate reductase |
|  |  | OIAAFOCL_01650 | 1,620,408 | 1,620,935 | 528 | - | Hypothetical protein |
|  |  | OIAAFOCL_01651 | 1,620,926 | 1,621,402 | 477 | - | Hypothetical protein |
|  |  | OIAAFOCL_01652 | 1,621,545 | 1,622,504 | 960 | + | HTH-type transcriptional activator RhaR |
|  |  | OIAAFOCL_01653 | 1,622,569 | 1,623,423 | 855 | - | Phospholipase YtpA |
|  |  | OIAAFOCL_01654 | 1,623,537 | 1,624,481 | 945 | - | Ferrochelatase |
|  |  | OIAAFOCL_01655 | 1,624,743 | 1,624,982 | 240 | - | Hypothetical protein |
|  |  | OIAAFOCL_01656 | 1,625,078 | 1,625,350 | 273 | - | 30S ribosomal protein S16 |
|  |  | OIAAFOCL_01657 | 1,625,439 | 1,626,650 | 1,212 | - | 3-hydroxy-3-methylglutaryl-coenzyme A reductase |
|  |  | OIAAFOCL_01658 | 1,626,752 | 1,627,891 | 1,140 | - | Acetyl-CoA acetyltransferase |
|  |  | **OIAAFOCL_01659** | **1,628,200** | **1,629,354** | **1,155** | **+** | **Hydroxymethylglutaryl-CoA synthase** |
|  |  | OIAAFOCL_01660 | 1,629,414 | 1,630,121 | 708 | - | Glucosamine-6-phosphate deaminase |
|  |  | OIAAFOCL_01661 | 1,630,272 | 1,631,231 | 960 | - | 2-succinyl-6-hydroxy-2,4-cyclohexadiene-1-carboxylate synthase |
|  |  | OIAAFOCL_01662 | 1,633,100 | 1,633,396 | 297 | - | Hypothetical protein |
|  |  | OIAAFOCL_01663 | 1,634,233 | 1,636,293 | 2,061 | + | Chromosome partition protein Smc |
|  |  | OIAAFOCL_01664 | 1,636,259 | 1,636,522 | 264 | + | Hypothetical protein |
|  |  | OIAAFOCL_01665 | 1,636,519 | 1,637,070 | 552 | + | Hypothetical protein |
|  |  | OIAAFOCL_01666 | 1,637,189 | 1,637,779 | 591 | + | Replication protein RepB |
|  |  | OIAAFOCL_01667 | 1,638,040 | 1,638,195 | 156 | + | Hypothetical protein |
|  |  | OIAAFOCL_01668 | 1,638,192 | 1,639,157 | 966 | + | Tyrosine recombinase XerC |
|  |  | OIAAFOCL_01669 | 1,639,301 | 1,640,338 | 1,038 | + | S-adenosylmethionine:tRNA ribosyltransferase-isomerase |
|  |  | OIAAFOCL_01670 | 1,640,414 | 1,640,725 | 312 | - | Hypothetical protein |
|  |  | OIAAFOCL_01671 | 1,640,804 | 1,641,976 | 1,173 | - | Hypothetical protein |
|  |  | OIAAFOCL_01672 | 1,642,099 | 1,646,955 | 4,857 | - | Hypothetical protein |
|  |  | OIAAFOCL_01673 | 1,646,992 | 1,647,747 | 756 | - | Hypothetical protein |
|  |  | OIAAFOCL_01674 | 1,647,785 | 1,648,543 | 759 | - | Hypothetical protein |
| SGL30088 | RIPP-like | DOPMHDDG_00613 | 604,172 | 604,345 | 174 | + | Hypothetical protein |
|  |  | DOPMHDDG_00614 | 604,384 | 604,557 | 174 | + | Hypothetical protein |
|  |  | DOPMHDDG_00615 | 604,831 | 605,070 | 240 | - | Hypothetical protein |
|  |  | DOPMHDDG_00616 | 605,617 | 605,814 | 198 | + | Hypothetical protein |
|  |  | DOPMHDDG_00617 | 606,176 | 606,982 | 807 | - | Accessory gene regulator protein A |
|  |  | DOPMHDDG_00618 | 606,987 | 608,150 | 1,164 | - | Hypothetical protein |
|  |  | DOPMHDDG_00619 | 608,156 | 608,284 | 129 | - | Hypothetical protein |
|  |  | DOPMHDDG_00620 | 608,474 | 608,590 | 117 | + | Hypothetical protein |
|  |  | **DOPMHDDG_00621** | **609,077** | **611,269** | **2,193** | **+** | **Lactococcin-G-processing and transport ATP-binding protein LagD** |
|  |  | DOPMHDDG_00622 | 611,280 | 612,659 | 1,380 | + | Lactococcin A secretion protein LcnD |
|  |  | DOPMHDDG_00623 | 612,833 | 613,057 | 225 | + | Hypothetical protein |
|  |  | DOPMHDDG_00624 | 613,306 | 613,566 | 261 | + | Hypothetical protein |
|  |  | DOPMHDDG_00625 | 613,872 | 615,227 | 1,356 | + | Hypothetical protein |
|  |  | DOPMHDDG_00626 | 615,328 | 615,576 | 249 | + | Hypothetical protein |
|  |  | DOPMHDDG_00627 | 615,815 | 616,099 | 285 | + | Hypothetical protein |
| SGL30089 | RIPP-like | AIKLKJDG_00613 | 604,172 | 604,345 | 174 | + | Hypothetical protein |
|  |  | AIKLKJDG_00614 | 604,384 | 604,557 | 174 | + | Hypothetical protein |
|  |  | AIKLKJDG_00615 | 604,831 | 605,070 | 240 | - | Hypothetical protein |
|  |  | AIKLKJDG_00616 | 605,617 | 605,814 | 198 | + | Hypothetical protein |
|  |  | AIKLKJDG_00617 | 606,176 | 606,982 | 807 | - | Accessory gene regulator protein A |
|  |  | AIKLKJDG_00618 | 606,987 | 608,150 | 1,164 | - | Hypothetical protein |
|  |  | AIKLKJDG_00619 | 608,156 | 608,284 | 129 | - | Hypothetical protein |
|  |  | AIKLKJDG_00620 | 608,474 | 608,590 | 117 | + | Hypothetical protein |
|  |  | **AIKLKJDG_00621** | **609,077** | **611,269** | **2,193** | **+** | **Lactococcin-G-processing and transport ATP-binding protein LagD** |
|  |  | AIKLKJDG_00622 | 611,280 | 612,659 | 1,380 | + | Lactococcin A secretion protein LcnD |
|  |  | AIKLKJDG_00623 | 612,833 | 613,057 | 225 | + | Hypothetical protein |
|  |  | AIKLKJDG_00624 | 613,306 | 613,566 | 261 | + | Hypothetical protein |
|  |  | AIKLKJDG_00625 | 613,872 | 615,227 | 1,356 | + | Hypothetical protein |
|  |  | AIKLKJDG_00626 | 615,328 | 615,576 | 249 | + | Hypothetical protein |
|  |  | AIKLKJDG_00627 | 615,815 | 616,099 | 285 | + | Hypothetical protein |


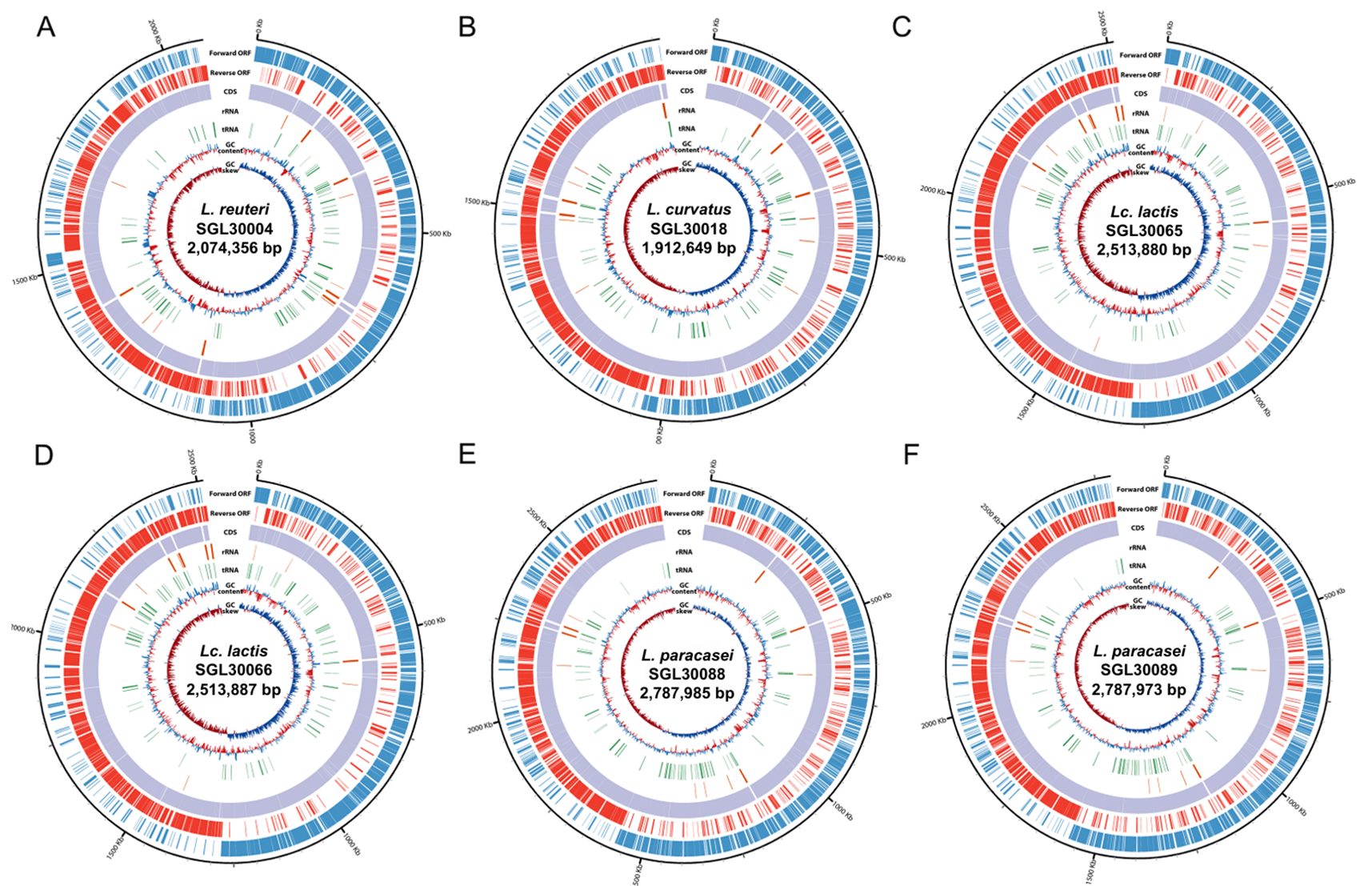


**Figure S1.** Circos genome maps of (A) *L. reuteri* SGL30004, (B) *L. curvatus* SGL30018, (C) *Lc. lactis* SGL30065, (D) *Lc. lactis* SGL30066, (E) *L. paracasei* SGL30088, and (F) *L. paracasei* SGL30089.


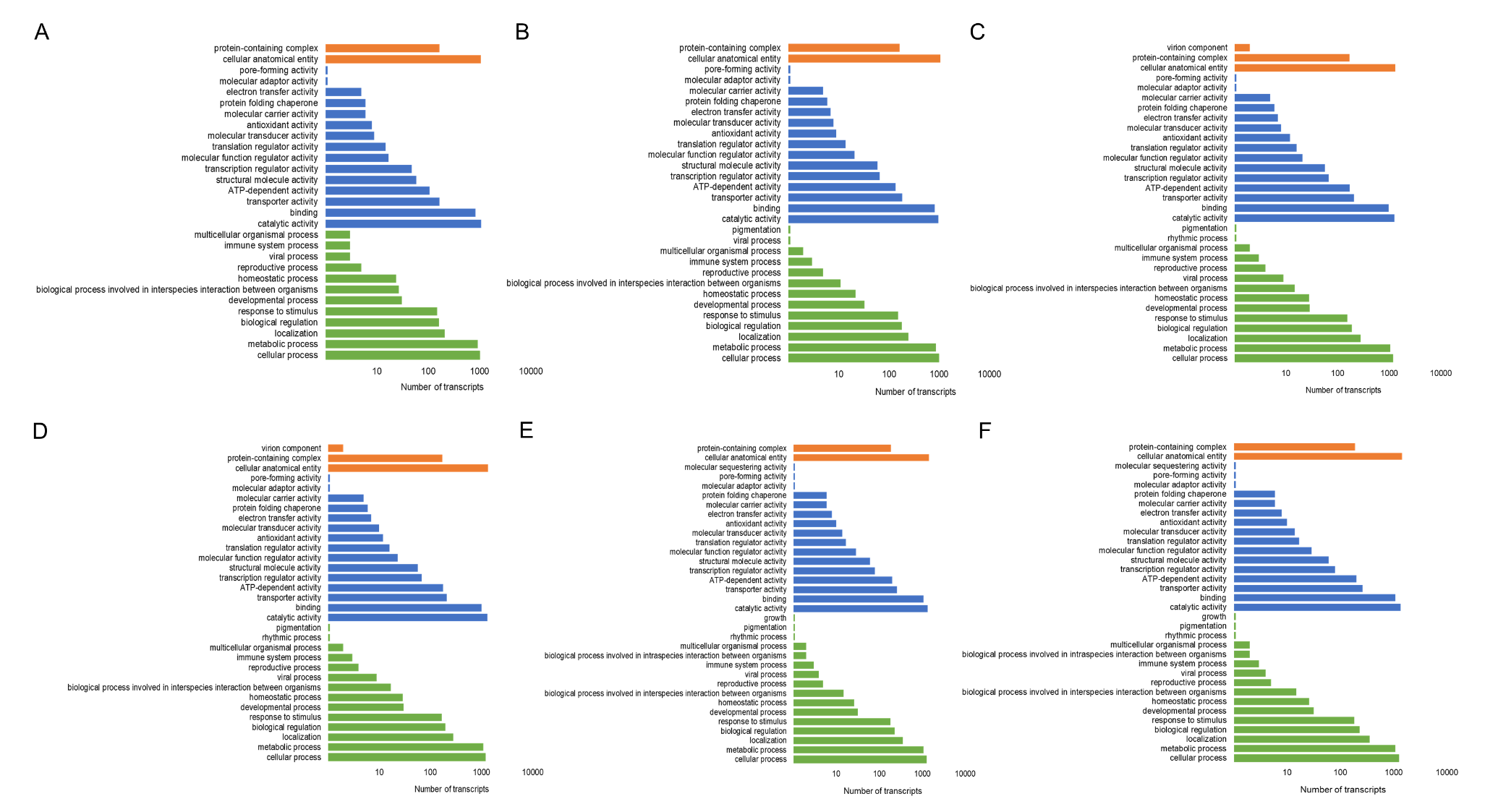


**Figure S2.** Gene Ontology (GO) graphs of (A) *L. reuteri* SGL30004, (B) *L. curvatus* SGL30018, (C) *Lc. lactis* SGL30065, (D) *Lc. lactis* SGL30066, (E) *L. paracasei* SGL30088, and (F) *L. paracasei* SGL30089.
